# Multi-resolution convolutional neural networks for inverse problems

**DOI:** 10.1038/s41598-020-62484-z

**Published:** 2020-03-31

**Authors:** Feng Wang, Alberto Eljarrat, Johannes Müller, Trond R. Henninen, Rolf Erni, Christoph T. Koch

**Affiliations:** 1 Electron Microscopy Center, Empa, Swiss Federal Laboratories for Materials Science and Technology, CH-8600 Dübendorf, Switzerland; 2 Institut für Physik, IRIS Adlershof der Humboldt-Universität zu Berlin, 12489 Berlin, Germany

**Keywords:** Materials science, Optics and photonics, Characterization and analytical techniques, Imaging techniques, Microscopy

## Abstract

Inverse problems in image processing, phase imaging, and computer vision often share the same structure of mapping input image(s) to output image(s) but are usually solved by different application-specific algorithms. Deep convolutional neural networks have shown great potential for highly variable tasks across many image-based domains, but sometimes can be challenging to train due to their internal non-linearity. We propose a novel, fast-converging neural network architecture capable of solving generic image(s)-to-image(s) inverse problems relevant to a diverse set of domains. We show this approach is useful in recovering wavefronts from direct intensity measurements, imaging objects from diffusely reflected images, and denoising scanning transmission electron microscopy images, just by using different training datasets. These successful applications demonstrate the proposed network to be an ideal candidate solving general inverse problems falling into the category of image(s)-to-image(s) translation.

## Introduction

Most physical theories allow us to make predictions: given a complete description of the state of a physical system, we can predict or simulate some measurements. Recovering parameters that describe the physical state of a system from measurement requires solving an inverse problem, which often can be a problem that cannot be solved deterministically and non-iteratively. The forward simulation is explicit, in many cases, much faster than any inversion, and may often also include more physically significant effects to the observed signal than most inverse algorithms. However, in many situations, physically meaningful information can only be extracted from experimental data by solving inverse problems, either by least-squares-minimization or probabilistic approaches, which often tend to be slow and probably under-determined due to a lack of data and their inner non-linearity. Inverse problems are some of the most important mathematical problems in science. However, in most cases, their solutions are designed to solve particular problems under particular conditions, making good use of domain-specific knowledge but lacking transferability to other inverse problems. The non-linear nature of many of these problems has so far prevented the framework of a generic solver.

Motivated by recent advances in the development of computing hardware and driven by large datasets^1,2^, deep learning^3^ has recently shown great potential particularly in image(s)-to-image(s) translation tasks, such as super resolution^4^, image denoising^5^ and image generation^6^. Many inverse applications^7,8^ have been purposed and have achieved promising results, since the universal approximation theorem guarantees that neural networks can approximate arbitrary functions well^9–11^. However, there are no guarantees those neural network weights can be found by optimization, and because of the high non-linearity of such deep architectures, their performance crucially depends on proper hyper-parameter configurations. Recent advanced big models even require years to develop and tens of thousands of dollars to train^12,13^. Deep learning has been widely used especially in recent computational imaging applications^14,15^, although many tricks exist to tune the hyper-parameters of a neural network such as the proper setting of the learning rate, the regulations, the design of the hidden units, the convolutional kernels and the network architectures^16,17^, none of these measures, achieves both a speed-up of convergence and quality of the resulting solution^18^.

**Figure 1:**
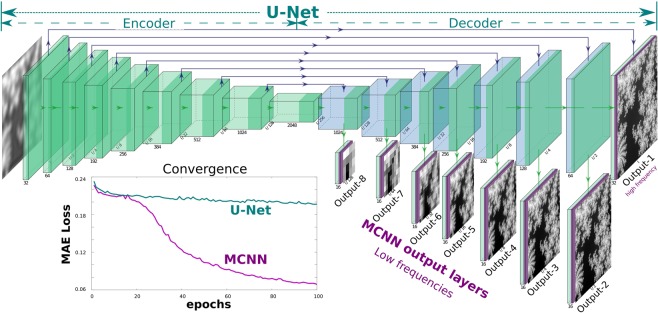
By matching outputs at all frequencies, MCNN achieves better stability and faster convergence than U-Net. A simplified architecture of an MCNN is demonstrated. This application is designed to predict phases from a defocused image. The network is composed of a classic U-Net (the upper part) with an additional 7 branches for multi-resolution reconstruction (from Output-2 to Output-8). With this topology, the input image is first encoded into a high dimension tensor, then is decoded into 8 images of different sizes to match different frequency components of the desired phases. The convergence curves of the test set show the significant advantages of an MCNN over a classic U-Net: the MAE of MCNN drops quickly in 100 iterations, while the U-Net converges slowly and can get stuck at local minima.

Aware of the fact that a deep convolutional neural network (DCNN) often first quickly recovers the dominant low-frequency components, and afterward the high-frequency ones in a rather slow manner^19,20^, and inspired by the idea of multi-grid methods^21^, we propose a novel multi-resolution deep convolutional neural network (MCNN). This architecture extends the functionality of the hidden layers in the decoder of a U-Net^22,23^ by connecting these hidden layers to additional convolution layers to produce coarse outputs, in an attempt to match the low-frequency components, as is demonstrated in Fig. 1. Further modification by attaching different coarse inputs to the layers in the encoder has also been tested, but no apparent improvements in convergence is observed. Another possible variation is mutating the architecture progressively by starting to fit only the components of low frequencies at the initial phases, then inserting new layers to match the increasingly high-frequency features during the training, as has been demonstrated in some of the recent applications^24,25^. This architecture speeds up the network convergence and dramatically stabilizes the training process. As is shown in the lower-left of Fig. 1, with identical setups, when solving an inverse Laplacian problem from a second-order gradient approximation, MCNN quickly reaches a mean-absolute-error (MAE) loss around 0.07 while the conventional U-Net is challenging to train, being trapped around 0.2. Additional normalization layers can also accelerate the convergence^26,27^, but will very likely introduce undesired distortions (extended data Fig. 2a,b), and should be used carefully. A topological setup of those coarse MCNN output branches are visualized in the lower right part of Fig. 1, in which the outputs matching the low-frequency components of different resolutions are shown; while the U-Net shown in the upper part directly matches the input layer to the desired output of the same resolution. More details, including the implementation, the training settings, and the prediction results, of this comparison, can be found within the notebook attached in the released code.

**Figure 2:**
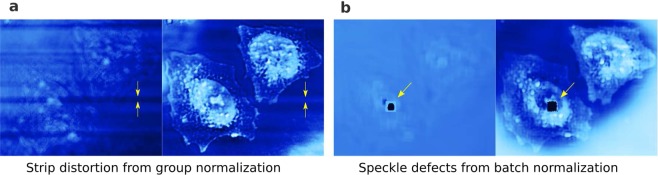
Extended Figure: Distortions introduced by normalization layers. Inserting normalization layers before or after the activation layers will improve the convergence of the networks, but further research is desired to deal with the distortions introduced by the normalization layers. This can be directly observed from the intermediary results when training to predict the phases and amplitudes from 8 defocused HeLa cell images. (**a**) Stripe distortion as a result of group normalization. (**b**) Speckle defects from batch normalization.

In contrast to conventional problem-oriented inverse applications, MCNN aims to solve every inverse problem falling into the category of image(s)-to-image(s) conversion, without being limited to specific applications, but relying on massive datasets from fast numerical simulation or direct measurement. This generalization capability is demonstrated by solving three different inverse problems.

## The phase problem

The first problem is to retrieve phases of a propagated complex wave by using the direct intensity measurements. This problem is a very fundamental inverse problem in optics, astronomy, or microscopy with neutrons, X-rays, or electrons. It has attracted a lot of research effort and led to the invention of numerous methods to reconstruct the missing phases from intensity measurements. Following Dennis Gabor’s proposal of the holographic principle^28^, numerous methods have been invented to extract phases by post-processing images^29–32^. These methods often-times only work under deliberate approximations or elaborate experimental configurations.

In conventional phase retrieval schemes, a trade-off between simple invertibility and accuracy has to be made. When approximating the imaging process by the transport of intensity equation (TIE)^29^, the relationship between the wavefront and a gradient measurement in the intensity domain can be approximated by a second-order partial differential equation, the solution of which can be found by assuming either periodic^33^ or other, often more appropriate boundary conditions^34^. To obtain a reliable gradient estimation, multiple intensities have to be measured at focal planes below and above the plane of focus, in pairs symmetrically to the in-focus plane^31,32^. One of the recent applications based on deep learning takes the complex wave of the back-propagated hologram intensities as inputs^35^, another variant retrieves the complex phase also making use of prior information from Fresnel propagation^36^, rather than directly mapping the measured intensities to desired phases. The proposed MCNN does not suffer from such restrictions. In our application, a *direct resolution* is demonstrated by mapping measurements to phases straightforwardly in an end-to-end manner. Phases can be predicted from intensities recorded at arbitrary plane(s) of focus. Since predicting the phase from its second-order gradient is a straight forward application, this problem has been included as a tutorial in our open-source codebase.

**Figure 3:**
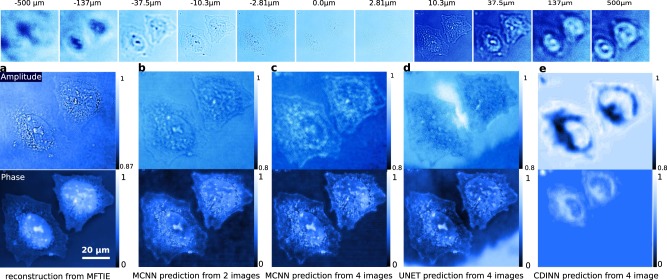
Amplitudes and phases predicted from defocused images. First row: 51 defocused Hela cell images recorded at focal planes exponentially spaced from  −500 *μm* to 500 *μm* (only 11 are shown). (**a**) Phases (top) and amplitudes (bottom) reconstructed by MFTIE from all 51 images^32^. A range-of-interest of 470 × 520 pixels is selected; (**b**) MCNN prediction using 2 images taken from  −1 *μm* and 1 *μm*; (**c**) MCNN prediction using 4 images taken from  −1.30 *μm* to 1.30 *μm*; (**d**) U-Net prediction using 4 images taken from  −1.30 *μm* to 1.30 *μm*; (**e**) CDINN prediction using 4 images taken from  − 1.30 *μm* to 1.30 *μm*.

The results are presented in Fig. 3, in which three models have been trained to predict the amplitudes and the phases of HeLa cells directly from recorded intensities. Comparing with the reference results shown in Fig. 3a, which is produced by multi-focus TIE (MFTIE)^32^ using all 51 images, these applications converge very quickly in 2 epochs with 2048 training samples, and yield, albeit predicted from different images, very similar phases, which are shown in Fig. 3b,c. The predicted amplitudes are quite blurred compared to the MFTIE result. And no apparent improvement has been observed by including more intensities and training even longer. This behavior is expected, since the neural networks are trained by minimizing the MAE, with which the optimizer will try to minimize the averaged error, giving equal weights to all pixels despite some pixels having particularly high errors. Other applications dealing with the pure phase case are also presented for a reference. In these applications, the prediction results from one and two measured intensities are given in extended Fig. 4b–d, which are quite similar to the result obtained by Gaussian process TIE (GPTIE) given in **a** for reference. It is worth mentioning that the conventional U-Net produces a worse result with identical settings of MCNN, as is shown in Fig. 3d, although it tends to give out similar results in the long run. Moreover, coherent diffraction imaging neural networks^37^ (CDINN) gives a rough contour, only reconstructing the low frequency components of the Hela cells, as is shown in Fig. 3e.

**Figure 4:**
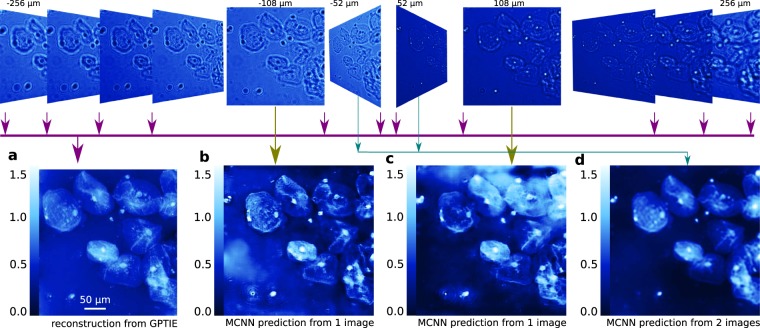
Extended Figure: Phase predicted from defocused image(s). From one or more defocused images of a pure phase object made up of human cheek cells acquired equally spaced by *d*_*z*_ = 4 *μm* from  −256 *μm* to 256 *μm* (only 11 shown), MCNNs are capable of phase prediction. (**a**) GPTIE reconstruction from gradients estimated using 129 images from different focal planes, with a range-of-interest of 945 × 888 pixels selected^31^. (**b**) Phase prediction from 1 image at a distance of  −108 *μm*, with 1024 × 1024 pixels. (**c**) Prediction from 1 image at a distance of 108 *μm*. (**d**) Prediction from 2 images at  −52 *μm* and 52 *μm*, showing good performance on par with the state-of-the-art reconstruction algorithm demonstrated in (**a**).

**Figure 5:**
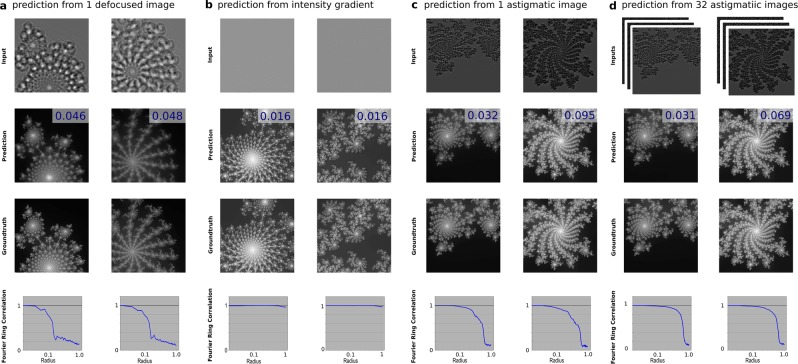
Extended Figure: MCNN gives good results in low-frequency domains. The simulated intensities are presented in the first row, and their predictions are presented in the second row (with their MAE shown in the upper right corner). The ground truths are presented in the third row for a visual comparison. The last row shows the Fourier ring correlations between the predictions and the ground truths. In (**a**), the inputs are defocused images, in which low-frequency details are prevalent. The low-frequency features recover well in this case. In (**b**), the inputs are intensity gradients, in which high-frequency details dominates. The high-frequency features are largely recovered in this experiment. In (**c,d**), the inputs are astigmatic images, simulated by rotating a cylinder lens with different angles. Multiple rotations can improve the quality of the output phases, as are shown in the last row of (**c**,**d**), but not so much in the high frequencies.

To further explore the prediction qualities in the frequency domain, the Fourier ring corrections (FRC) of several numerical experiments are calculated. Figure 5a shows that MCNN recovers low frequency components well from a single image. The high-frequency features can be compensated by introducing fine details in the inputs, as is shown in Fig. 5b. Including more measurements improves the performance, but not very apparent with the high frequencies, as is shown in Fig. 5c,d.

## Imaging objects from diffuse reflection

The second problem is to image objects that are hidden from direct view using their indirect diffuse light reflections. Observing objects located in inaccessible regions is particularly useful in the fields of remote sensing, computer vision, and autonomous driving. This problem has drawn significant attention in recent publications^38–40^, but most of these applications require controlled or time-varying illumination, high-speed sensing or complicated inversion algorithms employing ray optics.

Although deep learning has been employed to do object classification from non-line-of-sight (NLOS) imaging^41^, our application demonstrates experimentally for the first time, to our best knowledge, that a series of colorful two-dimensional objects can be predicted from DNN.

**Figure 6:**
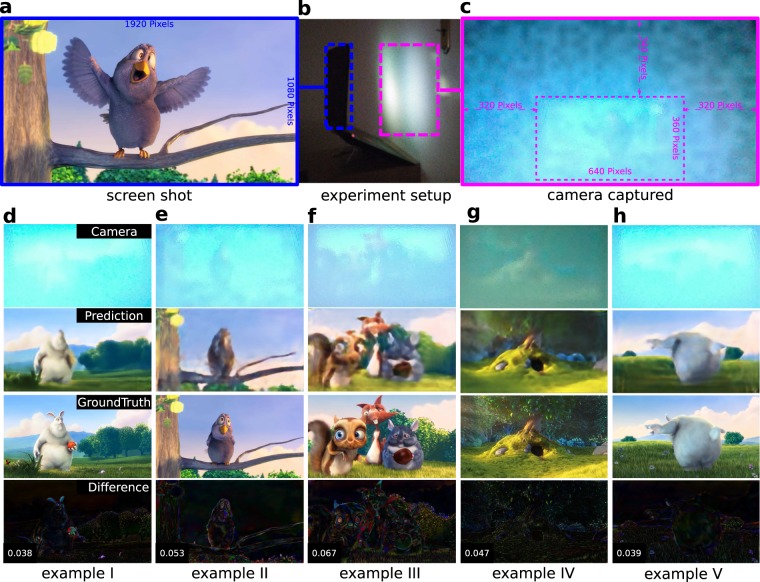
Imaging objects from diffuse reflections. This is done by training an MCNN matching the camera captured diffuse reflective images to the screenshot images. (**a**) A screenshot of the video *Big Buck Bunny*^42^ being displayed. This is one of the target images matching the MCNN output. (**b**) Experimental setup. Everything is done with a laptop facing a door, no additional devices required. (**c**) A camera captured image corresponding to the screenshot shown in (**a**). The cropped zone marked with a dotted rectangle is one of the input images of the MCNN. (**d**–**h**) Predictions randomly sampled from test set. The first row shows the selected range of the camera captured images (cropped and flipped), the second row shows the predictions, the third row shows the screenshots and the last row shows the absolute difference between the predictions and the screenshots, with the MAE value presented at the lower-left corner.

The training of our neural networks relies on the massive dataset. The dataset can be simulated from existing theories if the domain-specific knowledge is well-established. In cases of simulations that are not feasible due to theory or unknown parameters, the training dataset can also be collected from direct measurements. Using an ordinary laptop, with 768 images captured by its camera and 768 screenshots recorded from its screen, our MCNN could be trained and enabled to reconstruct additional screenshots from diffusely reflected rays, i.e., the camera-captured images. The data collection procedure is shown in **b** of Fig. 6: a laptop is facing a door with a program running on it to record images from the screen and the camera at two frames-per-second (fps). One pair of the captured images are shown in Fig. 6a,c. The predicted images from the testing set match the ground truth well for images dominated by low-frequency features. The prediction of the prediction can be verified from the visualized absolute difference shown at the last row in Fig. 6d–h, where most differences are close to zero (black), especially the regions corresponding to the sky, the cloud, and the green grass. Nevertheless, the high-frequency details are missing in the predicted images as the optimizer is minimizing MAE. For example, in Fig. 6d, the ear of the rabbit and the red apple are lost; in **e** and **g**, the details of the eyes are lost; in **f**, all details of the animals are blurry. More results on the test set are included in the extended data video 1.

## Denoising STEM images

The third problem is to denoise heavily noisy scanning transmission electron microscopy (STEM) images. Modern STEM can provide sub-ångström imaging resolution^43,44^, but this is limited by, e.g, the beam sensitivity of the specimen. Lowering the electron dose results in noisy images with a poor signal-to-noise ratio (SNR) less than 0 dB, and complicated extraction of relevant specimen information. Moreover, STEMs can produce millions of images in a few hours at a speed of 100 fps, and this amount of data can require months to process for a conventional algorithm. It is, therefore, essential to predict realistic images from noisy observations very quickly without loss of information^45^. Due to the complex unknown environmental variables of specific STEM setups, real-world STEM image denoising is too complicated for a single monolithic denoising algorithm. Many conventional algorithms exist to address this problem, but they heavily depend on domain-specific knowledge or *a priori* information^46,47^, and their application for real-time denoising is difficult. Recent variations^48–53^ based on DCNNs work well by directly matching a noisy input image to a clean output image, or using unclean images or unpaired images at a price of a small performance penalty^54–56^, but they are constrained to high SNR images, mostly above 10.0 dB, with known noise sources, and most of them only consider a single signal independent noise with known levels.

**Figure 7:**
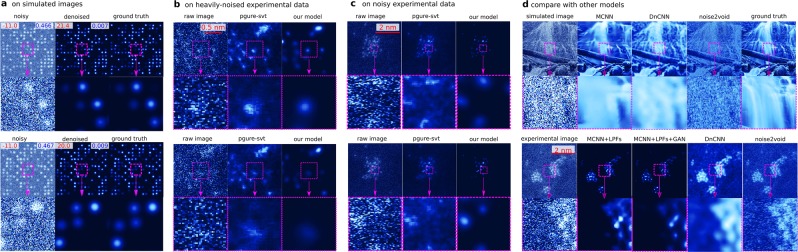
Validation of the denoising application on heavily-noised aberration-corrected high-annular dark-field (HAADF) STEM images of sub-nanometre sized Platinum clusters. MCNN performs well on heavily-noised datasets, as is demonstrated in (**a**). The numbers on the upper-left corner are the SNRs and the upper-right are MAEs. Also MCNN gives out clear and consistent results on consecutive experimental image frames shown in the left columns, which are recorded at 150 fps with 128  ×  128 pixels (**b**) and at 15 fps with 512  ×  512 pixels (**c**), and are taken under electron dose in range [10^5^, 10^6^]eÅ^−2^*s*^−1^ with a FEI Titan Themis. The upper images are for the first frames, the lower images are for the second frames. Similar denoising results produced by PGURE-SVT are shown in the middle columns for a comparison, which are not as clear as the MCNN results in the right columns. The neural network without the LPFs layer can still predict clear result, if the input images have the same noisy features, as is shown in the upper rows of (**d**); but when applying this model to experimental images, the neural network equipped with the LPFs layer gives much better result than conventional neural network such as the recent noise2void model and DnCNN model. Fine-running the model with the LPFs layer by connecting a conditional generative adversarial network (GAN), the clusters in the predicted image are more atomic-like, as is shown in lower rows of (**d**).

**Figure 8:**
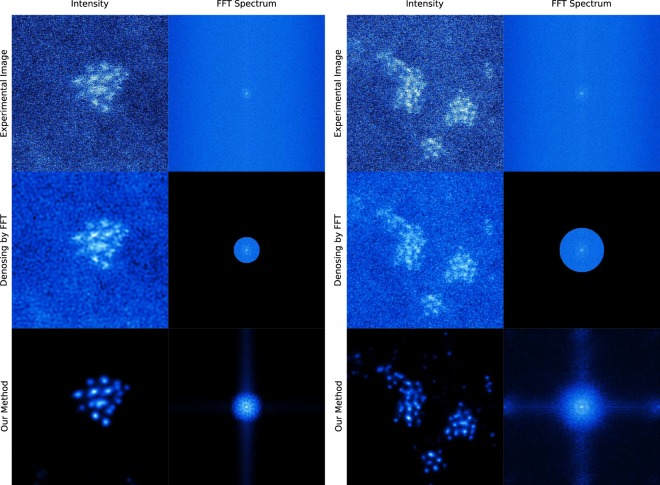
Extended Figure: Denoising behavior in frequency domain. Our method gives much more Gaussian-like results than conventional denoising method using a low pass filter. Our method identifies most of the high frequency components as noises, and also modifies the low frequency components in pursuit of clear atomic images.

Here we train our MCNN to recover clean results from massively noisy STEM images recorded with less than ten counts per pixel, falling into an SNR less than 0 dB, which means that our experimental data contain more noises than signals. If all the noise parameters are known, a training set can be simulated according to these parameters. With such a training set, a neural network can yield a good prediction, as is shown in Fig. 7d. However, since various noises of unknown levels exist in the recorded STEM images, including but not limited to the Poisson noise, Gaussian noise, and clipping noise, it is nearly impossible to simulate a proper training set. The first layer of our neural network is manually designed to mimic the performance of four LPFs to address this problem. This strategy can be understood as converting the denoising problem into a deblurring problem. With much deeper architecture and much more trainable parameters, this model gives out clearer results than the most recent noise2void model^56^ and the well-known residual Gaussian denoising convolution neural networks^5^ (DnCNN), as is demonstrated in the lower rows in Fig. 7d. To further study the behavior of our model, the frequency responses of two randomly selected denoised HAADF images acquired at different conditions are presented in Extend Fig. 8a,b. While identifying most of the high-frequency components of the experimental images as noises, our model effectively modulates the low-frequency components as well, predicting much more Gaussian-like images than conventional denoising methods using a low pass filter. Our model gives a Gaussian-like shape for atomic peaks, which is to be expected as the experimental images are formed by a Gaussian-like electron beam being scanned (convolved) over sub-pixel sized atomic nuclei. Our network achieves an excellent performance of up to 440 fps when working with images taken at 150 fps with 128 × 128 pixels, more than three orders of magnitude faster than conventional methods, taking significant advantage of modern hardware acceleration like other deep learning applications (extended data Fig. 9).

**Figure 9:**
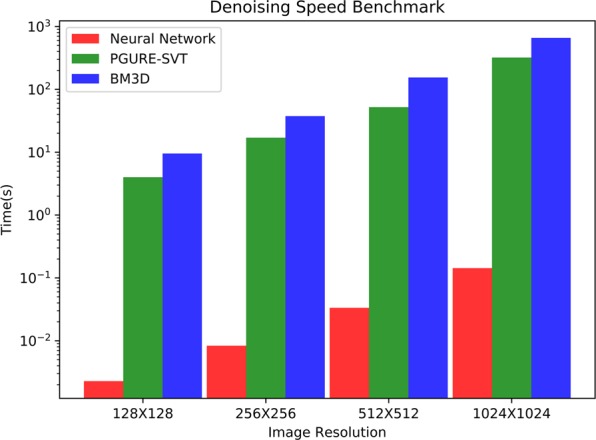
Extended Figure: MCNN outperforms conventional denoising algorithms by more than three orders. This figure shows the average denoising time for MCNN, BM3D and PGURE-SVT methods, on experimental images from 128 × 128 pixels to 1024 × 1024 pixels.

The effectiveness of this MCNN application has been validated in two ways. First, it is tested using simulated noisy atomic images with Poisson noise, Gaussian noise, and white noise of SNR less than  −10 dB. In this task, the MCNN achieves an average SNR as high as 20 dB. Two examples are shown in Fig. 7a, in which all atoms are successfully restored, and the MAEs are less than 0.01. Second, it is cross-validated with the state-of-the-art Poisson-Gaussian Unbiased Risk Estimator for Singular Value Thresholding (PGURE-SVT) algorithm^47^. Two randomly selected consecutive frames from two experimental dataset are shown in Fig. 7b,c. Visually comparing their denoised results, the neural network gives similar, but much clearer atomic images on consecutive frames, demonstrating the effectiveness of MCNN, despite the predictions are only from single frames. This result allows for studying the actual dynamics of the atoms in real time. More results on the experimental dataset can be found in the extended data video 2 and 3.

**Figure 10:**
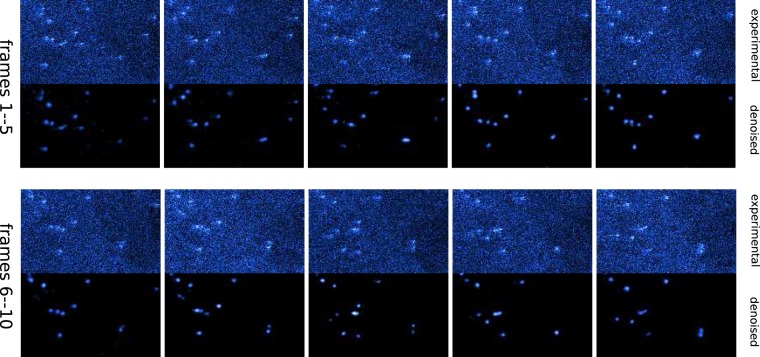
Extended Figure: Denoising HAADF images with coma distortions. MCNN gives clear and consistent results on consecutive frames containing coma distortions, even though our model has never seen this kind of data before.

Additionally, our model gives clear and consistent Gaussian-like results on consecutive frames suffering from coma defects, as is shown in extend Fig. 10. This probably due to the hand-crafted LPFs in the first layer which effectively removes this kind of error, even though our model has never been trained on such data.

## Conclusion

An application-neutral framework for solving inverse problems in different domains that involve image to image mappings has been proposed and demonstrated. The generic capability of this framework has been demonstrated using three applications in very different domains. The difficulties of a challenging application, the complex experimental setups, and the complicated inverse algorithm implementation has been alleviated with this framework. Quick and smooth convergence is guaranteed by matching additional output layers to corresponding low-frequency features, reducing the frustration of DNN hyper-parameter tuning, providing sufficient datasets available either from numerical simulation or direct measurement. We expect that this robust, general-purpose architecture, will inspire the emergence of a new branch of new schemes that deal with different kinds of inverse problems, broadening the scope of inverse problem applications.

## Method

### Dataset

For phase retrieval applications, we simulated the training sets using Fourier optics^57^, including slight Poison noise and white noise. We randomly sampled the phases and amplitudes from Open Images Dataset^58^ (OID). Then we simulated millions of defocused images. However, for each MCNN, only 2,048 random samples were selected to train, as we did not find apparent performance improvement when expanding the training set even to 51,200. The exponentially spaced defocused images shown in Fig. 4 come from an open access GPTIE dataset^31^. Each image has 1024 × 1024 pixels with effective size 0.31 × 0.31 *μm*^2^ per pixel. The sample is unstained cheek cells from a human’s mouth, placed on a microscope slide and sealed with a cover slide. This sample represents nearly a pure phase object, though there are some specks of amplitude variation. The defocused images shown in Fig. 3 come from the MFTIE dataset^32^. Each image has 2560 × 2160 pixels with effective size 0.1625 × 0.1625 *μm*^2^ per pixel. The sample is HeLa cancer cells, which are relatively transparent.

For the diffused reflection reconstruction application, we collected the dataset using an HP OMEN 17 laptop. With a full-screened film, *Big Buck Bunny*^42^ with 4K resolution and 60 Hz frame-rate, being played at a half-speed on a 17.3 inches LCD screen (16:9, 43.9 cm in diagonal), this laptop, mounted on a laptop stand, was positioned facing to a door at a distance about 30 cm, as is shown in Fig. 6 (B), and a Python script was running simultaneously, acquiring pictures (RGB) from its camera with a resolution of 1280 × 720 pixels and capturing screenshot images (RGB) with a resolution of 1920 × 1280 pixels at 2 fps. Multiple sources of noise exist in the acquired image-pairs coming from three main contributors. Firstly, the sensitivity of the camera was adjusted automatically and adaptively, resulting in slightly varying brightness and contrast between frames. This is very apparent in the first few frames. Secondly, the laptop vibrated a lot during recording. A powerful gaming GPU is equipped on this laptop, and a loud fan is attached to the GPU for thermal control. This GPU was under heavy pressure when a 4K resolution film was displaying at a screen with a refresh rate of 120 Hz, and the fan switched its working mode frequently during the acquisition time. Lastly, there was a small but random time interval between the screen-shooting and camera capturing. The acquisition program is coded carefully in a way ensuring both the screen images and the camera images are cached in the random access memory (RAM) before saving to the hard disk, but still, the Python script runs slowly in nature and its garbage collection behavior is uncontrollable, and therefore the uncertainty of the timing difference is inevitable. After 1024 image pairs were collected, we selected the first 256 pairs for testing, and the rest 768 pairs for training by matching the images from the camera to the screenshots. We cropped an area of 640 × 360 pixels of each input image to fit our model into the GPU model, and the output images were scaled from 1920 × 1280 pixels down to 1280 × 720 pixels. Besides, as a mirror reflect an image with a left/right reversal, the cropped input images were horizontally flipped. We presented all the predicted results from the first 256 camera captured images in the extended data video 1.

For the denoising application, we included Poisson noise, Gaussian noise, white noise, and clipping noise in the training set generation. We generated millions of images from the clear images randomly sampled from OID images and simulated annular dark-field (ADF) STEM images. These ADF images are simulated using convolutions between random pulse signals and 2D Gaussian atomic peaks, with 512 × 512 pixels. For the first training stage, we trained the neural network with half of the images from OID and the other half from ADF images. It is crucial to include random images sampled from OID. This strategy prevents the network from predicting everything to be zero at the very beginning because the simulated ADF images contain small average intensities. For the second stage, we fine-tuned this neural network on millions of simulated ADF images with a minimal learning rate. Later we re-tuned this neural network with millions of atomic images simulated from molecular dynamics using up to 14 atoms with 128 × 128 pixels but did not observe an apparent difference in the trained denoising model. We tested the denoising network on various experimental STEM images recorded using a probe corrected FEI Titan Themis at 300 kV, with an electron dose ranging from 10^5^*e*Å^−2^ to 10^6^*e*Å^−2^. The samples were made by plasma sputtering Pt onto Protochips Fusion thermal chips. The typical pixel sizes of the recorded images are 6.3–12.5 pm acquired at 15-150 fps and resolutions from 128 × 128 pixels to 512 × 512 pixels.

### Network architectures

The convolution layers inside each cell shown in Fig. 1 and the depth of the network are flexible of design but are generally restricted by the hardware and dataset. In our applications, we tested two types of architectures commonly used in recent deep learning applications. For the first type, there is a single convolutional layer with stride 2 in the encoder and a single deconvolutional layer with stride 2 in the decoder. This is the choice of the phase-only retrieval shown in Fig. 4 and the denoising application shown in Fig. 7, with about 50 million trainable parameters inside. For the phase-and-amplitude retrieval application shown in Fig. 3 and diffuse reconstruction application shown in Fig. 6, a bottleneck structure is selected in each of the unit cells, expanding the network to hundreds of layers, but reducing the trainable parameters to 20 million, to accelerate the training process and fit the model into the GPU memory. This is inspired by the recent architectures ResNeXt^59^ and Xception^60^. Also, the low-frequency output branches are reduced from 7 to 3 to fit everything into GPU memory. For the denoising network, an extra four-channeled layer is inserted right after the input layer. The filters of this layer are specially designed to mimic the functionality of 4 LPFs:

1 $$\begin{array}{ll}{f}_{1}(r,c)=1 & \ r,c\in [1,5],\\ {f}_{2}(r,c)=1 & \ r,c\in [1,7],\\ {f}_{3}(r,c)={e}^{-[{(r-8)}^{2}+{(c-8)}^{2}]{\rm{/}}\sqrt{20}} & \ r,c\in [1,15],\\ {f}_{4}(r,c)={e}^{-[{(r-8)}^{2}+{(c-8)}^{2}]{\rm{/}}\sqrt{30}} & \ r,c\in [1,15],\end{array}$$

in which *r* and *c* are the row and column index of the filters. This hand-crafted layer significantly stabilized the performance of the neural network, enabling it to provide robust prediction when the noise levels vary from images to images as the experimental setup changes with time. Furthermore, to prevent the network from predicting every pixel to be zero during the training and to make the high-intensity pixel clusters more atomic-like, a GAN in the conditional setting has been employed to adjust the back-propagated errors^61^. The different prediction behaviours of MCNN, MCNN with LPFs, and MCNN with LPFs and GAN are demonstrated in Fig. 7d.

### Training settings

We trained all the networks with 2 Nvidia GTX 1080 Ti GPUs using an Adam optimizer^62^. The phase retrieval applications converge very quickly, and we trained all these networks two epochs with a batch size of 4 in a few hours. We trained the diffuse reflection reconstruction application for 256 epochs with a batch size of 2 in a few days. For the denoising model, we pre-trained it eight epochs without attaching a GAN, and then we constructed a U-Net using the extracted weights from the corresponding MCNN layers. We fine-tuned this U-Net model by connecting an additional GAN with a tiny learning rate for 4096 epochs with a batch size of 8. The total training time is about one week. More detailed training settings are available within the released source code.

## Supplementary information

Supplementary Video1.

Supplementary Video2.

Supplementary Video3.

Supplementary Information.

## Data Availability

An open-source version is available at https://github.com/fengwang/MCNN. Related datasets and a minimal MCNN tutorial will found in this repository as well. An demo project to reproduce the results in this paper is a at https://github.com/fengwang/mcnn-demo, and an online capsule is available at https://codeocean.com/capsule/6012862/tree/v1.

## References

[1] Russakovsky O (2015). Imagenet large scale visual recognition challenge. International Journal of Computer Vision.

[2] Krasin, I.*et al*. Openimages: A public dataset for large-scale multi-label and multi-class image classification. **2**, 7 (2016).

[3] LeCun Y, Bengio Y, Hinton G (2015). Deep learning. Nature.

[4] Dong C, Loy CC, He K, Tang X (2016). Image super-resolution using deep convolutional networks. IEEE Transactions on Pattern Analysis and Machine Intelligence.

[5] Zhang K, Zuo W, Chen Y, Meng D, Zhang L (2017). Beyond a Gaussian Denoiser: Residual Learning of Deep CNN for Image Denoising. IEEE Transactions on Image Processing.

[6] Isola, P., Zhu, J.-Y., Zhou, T. & Efros, A. A. Image-to-image translation with conditional adversarial networks. *CVPR* (2017).

[7] Hezaveh YD, Levasseur LP, Marshall PJ (2017). Fast automated analysis of strong gravitational lenses with convolutional neural networks. Nature.

[8] Sinha A, Lee J, Li S, Barbastathis G (2017). Lensless computational imaging through deep learning. Optica.

[9] Hornik K, Stinchcombe M, White H (1989). Multilayer feedforward networks are universal approximators. Neural Networks.

[10] Cybenko G (1989). Approximation by superpositions of a sigmoidal function. Mathematics of Control, Signals and Systems.

[11] Lu, Z., Pu, H., Wang, F., Hu, Z. & Wang, L. The Expressive Power of Neural Networks: A View from the Width. In Guyon, I. *et al*. (eds.) *Advances in Neural Information Processing Systems*** 30**, 6231–6239 (Curran Associates, Inc., 2017).

[12] Yang, Z. *et al*. XLNet: Generalized Autoregressive Pretraining for Language Understanding. *arXiv:1906.08237* (2019).

[13] Brock, A., Donahue, J. & Simonyan, K. Large Scale GAN Training for High Fidelity Natural Image Synthesis. *arXiv:1809.11096* (2018).

[14] Barbastathis, G., Ozcan, A. & Situ, G. On the use of deep learning for computational imaging **6**, 921–943, https://www.osapublishing.org/optica/abstract.cfm?uri=optica-6-8-921.

[15] Jin, K. H., McCann, M. T., Froustey, E. & Unser, M. Deep Convolutional Neural Network for Inverse Problems in Imaging. In *IEEE Transactions on Image Processing*, **26**, 4509–4522, 10.1109/TIP.2017.2713099 (2017). 10.1109/TIP.2017.2713099 28641250

[16] Luo, R., Tian, F., Qin, T., Chen, E. & Liu, T.-Y. Neural architecture optimization. In Bengio, S. *et al*. (eds.) *Advances in Neural Information Processing Systems 31*, 7816–7827 (Curran Associates, Inc., 2018).

[17] Jin, H., Song, Q. & Hu, X. Auto-keras: An efficient neural architecture search system. In *Proceedings of the 25th ACM SIGKDD International Conference on Knowledge Discovery & Data Mining*, 1946–1956 (ACM, 2019).

[18] Smith, L. N. A disciplined approach to neural network hyper-parameters: Part 1 - learning rate, batch size, momentum, and weight decay. *arXiv:1803.09820* (2018).

[19] Xu, Z.-Q. J., Zhang, Y. & Xiao, Y. Training behavior of deep neural network in frequency domain. *arXiv preprint arXiv:1807.01251* (2018).

[20] Rahaman, N. *et al*. On the Spectral Bias of Deep Neural Networks. *arXiv preprint arXiv:1806.08734* (2018).

[21] Stüben, K. & Trottenberg, U. Multigrid methods: Fundamental algorithms, model problem analysis and applications. In *Multigrid Methods*, 1–176 (Springer, 1982).

[22] Hinton GE, Salakhutdinov RR (2006). Reducing the dimensionality of data with neural networks. science.

[23] Ronneberger, O., Fischer, P. & Brox, T. U-net: Convolutional networks for biomedical image segmentation. In *International Conference on Medical Image Computing and Computer-Assisted Intervention*, 234–241 (Springer International Publishing, 2015).

[24] Karras, T., Aila, T., Laine, S. & Lehtinen, J. Progressive Growing of GANs for Improved Quality, Stability, and Variation. *arXiv:1710.10196* (2017).

[25] Nah, S., Kim, T. H. & Lee, K. M. Deep multi-scale convolutional neural network for dynamic scene deblurring. In *The IEEE Conference on Computer Vision and Pattern Recognition (CVPR)* (2017).

[26] Ioffe, S. & Szegedy, C. Batch normalization: Accelerating deep network training by reducing internal covariate shift. In *Proceedings of the 32Nd International Conference on International Conference on Machine Learning - Volume 37*, ICML’15, 448–456 (JMLR.org, 2015).

[27] Wu, Y. & He, K. Group normalization. In *Proceedings of the European Conference on Computer Vision (ECCV)*, 3–19 (2018).

[28] Gabor D (1948). A New Microscopic Principle. Nature.

[29] Teague MR (1983). Deterministic phase retrieval: A Green’s function solution. JOSA.

[30] Van Dyck, D., Op de Beeck, M. & Coene, W. Object wavefunction reconstruction in high resolution electron microscopy. In *Proceedings of 1st International Conference on Image Processing*, vol. 3, 295–298 vol.3 (1994).

[31] Jingshan Z, Claus RA, Dauwels J, Tian L, Waller L (2014). Transport of Intensity phase imaging by intensity spectrum fitting of exponentially spaced defocus planes. Optics Express.

[32] Eljarrat A, Müller J, Huang MRS, Koch CT (2018). Multi-focus TIE algorithm including partial spatial coherence and overlapping filters. Optics Express.

[33] Nugent KA, Gureyev TE, Cookson D, Paganin D, Barnea Z (1996). Quantitative phase imaging using hard x-rays. Phys. Rev. Lett..

[34] Parvizi A, Van den Broek W, Koch CT (2017). Recovering low spatial frequencies in wavefront sensing based on intensity measurements. Advanced Structural and Chemical Imaging.

[35] Rivenson Y, Zhang Y, Günaydin H, Teng D, Ozcan A (2018). Phase recovery and holographic image reconstruction using deep learning in neural networks. Light: Science & Applications.

[36] Goy A, Arthur K, Li S, Barbastathis G (2018). Low Photon Count Phase Retrieval Using Deep Learning. Physical Review Letters.

[37] Cherukara, M. J., Nashed, Y. S. G. & Harder, R. J. Real-time coherent diffraction inversion using deep generative networks. *Scientific Reports* **8**, 16520, http://www.nature.com/articles/s41598-018-34525-1 (2018). 10.1038/s41598-018-34525-1 PMC6224523 30410034

[38] Klein J, Peters C, Martìn J, Laurenzis M, Hullin MB (2016). Tracking objects outside the line of sight using 2d intensity images. Scientific Reports.

[39] O’Toole M, Lindell DB, Wetzstein G (2018). Confocal non-line-of-sight imaging based on the light-cone transform. Nature.

[40] Saunders C, Murray-Bruce J, Goyal VK (2019). Computational periscopy with an ordinary digital camera. Nature.

[41] Satat, G., Tancik, M., Gupta, O., Heshmat, B. & Raskar, R. Object classification through scattering media with deep learning on time resolved measurement. *Optics Express* **25**, 17466–17479, https://www.osapublishing.org/oe/abstract.cfm?uri=oe-25-15-17466 (2017). 10.1364/OE.25.017466 28789238

[42] Blender Foundation. Big buck bunny, http://bigbuckbunny.org/ (2008).

[43] Erni R, Rossell MD, Kisielowski C, Dahmen U (2009). Atomic-resolution imaging with a sub-50-pm electron probe. Physical review letters.

[44] Jiang Y (2018). Electron ptychography of 2D materials to deep sub-Ångström resolution. Nature.

[45] Henninen, T. R., Bon, M., Wang, F., Passerone, D. & Erni, R. The Structure of Sub-nm Platinum Clusters at Elevated Temperatures. *Angewandte Chemie International Edition*, 10.1002/anie.201911068 (2019). 10.1002/anie.201911068 31682061

[46] Mevenkamp, N. *et al*. Poisson noise removal from high-resolution STEM images based on periodic block matching. *Advanced Structural and Chemical Imaging* **1**, 3 (2015-03-25).

[47] Furnival T, Leary RK, Midgley PA (2017). Denoising time-resolved microscopy image sequences with singular value thresholding. Ultramicroscopy.

[48] Guo, S., Yan, Z., Zhang, K., Zuo, W. & Zhang, L. Toward convolutional blind denoising of real photographs. In *Proceedings of the IEEE Conference on Computer Vision and Pattern Recognition*, 1712–1722 (2019).

[49] Lefkimmiatis, S. Universal denoising networks: a novel CNN architecture for image denoising. In *Proceedings of the IEEE conference on computer vision and pattern recognition*, 3204–3213 (2018).

[50] Ulyanov, D., Vedaldi, A. & Lempitsky, V. Deep image prior. In *Proceedings of the IEEE Conference on Computer Vision and Pattern Recognition*, 9446–9454 (2018).

[51] Wang, P., Zhang, H. & Patel, V. M. SAR Image Despeckling Using a Convolutional Neural Network. *IEEE Signal Processing Letters* **24**, 1763–1767, http://ieeexplore.ieee.org/document/8053792/ (2017).

[52] Hasinoff SW (2016). Burst photography for high dynamic range and low-light imaging on mobile cameras. ACM Transactions on Graphics (TOG).

[53] Xie, J., Xu, L. & Chen, E. Image Denoising and Inpainting with Deep Neural Networks. In Pereira, F., Burges, C. J. C., Bottou, L. & Weinberger, K. Q. (eds.) *Advances in Neural Information Processing Systems* **25**, 341–349 (Curran Associates, Inc., 2012).

[54] Lehtinen, J. *et al*. Noise2noise: Learning Image Restoration without Clean Data. *arXiv preprint arXiv:1803.04189* (2018).

[55] Batson, J. & Royer, L. Noise2self: Blind Denoising by Self-Supervision. *arXiv:1901.11365 [cs, stat]*, http://arxiv.org/abs/1901.11365. ArXiv: 1901.11365 (2019).

[56] Krull, A., Buchholz, T.-O. & Jug, F. Noise2void-learning denoising from single noisy images. In *Proceedings of the IEEE Conference on Computer Vision and Pattern Recognition*, 2129–2137 (2019).

[57] Eljarrat, A. Python code for inline holography using through-focus image series, https://github.com/AEljarrat/inline_holo (2018).

[58] Krasin, I. *et al*. Openimages: A public dataset for large-scale multi-label and multi-class image classification. *Dataset available from*, https://github.com/openimages **2**, 3 (2017).

[59] Xie, S., Girshick, R., Dollàr, P., Tu, Z. & He, K. Aggregated residual transformations for deep neural networks. In *Proceedings of the IEEE conference on computer vision and pattern recognition*, 1492–1500 (2017).

[60] Chollet, F. Xception: Deep Learning with Depthwise Separable Convolutions. In *2017 IEEE Conference on Computer Vision and Pattern Recognition (CVPR)*, 1800–1807 (IEEE, Honolulu, HI, 2017).

[61] Goodfellow, I. *et al*. Generative adversarial nets. In Ghahramani, Z., Welling, M., Cortes, C., Lawrence, N. D. & Weinberger, K. Q. (eds.) *Advances in Neural Information Processing Systems* **27**, 2672–2680 (Curran Associates, Inc., 2014).

[62] Kingma, D. P. & Ba, J. Adam: A method for stochastic optimization. In *3rd International Conference on Learning Representations, ICLR 2015, San Diego, CA, USA, May 7–9, 2015, Conference Track Proceedings*, http://arxiv.org/abs/1412.6980 (2015).

