# Supplementary material for: Multi-resolution convolutional neural networks for inverse problems
Source: Sci Rep. 2020 Mar 31;10:5730. doi: 10.1038/s41598-020-62484-z (PMC7109091; doi:10.1038/s41598-020-62484-z)
Supplement: Supplementary file 4 — Supplementary Information. [file 41598_2020_62484_MOESM4_ESM.pdf]

A minimal MCNN example

Given an image of Lenna, we can simulate its second gradient with a Laplace operator.

One of the ways for the simulation is an image convolution with a discrete kernel

$$\begin{vmatrix} 1 & 2 & 1 \\ 2 & -12 & 2 \\ 1 & 2 & 1 \end{vmatrix}$$

This is straightforward and easy

In [1]:
```
%matplotlib inline
import matplotlib
import matplotlib.pyplot as plt
plt.rcParams["figure.figsize"] = (20,10)

import tifffile
ground_truth = tifffile.imread( '../data/lena.tif')

import numpy as np
kernel = np.asarray( [[1, 2, 1], [2, -12, 2], [1, 2, 1]], dtype='float32' )
ground_truth = (ground_truth-np.amin(ground_truth))/(np.amax(ground_truth)-np.amin(ground_truth))

from scipy.signal import convolve
gradient = convolve( ground_truth, kernel, mode='same' )

fig, axs = plt.subplots(1, 2)
axs[0].set_title('gradient')
axs[0].imshow(gradient)
axs[1].set_title('ground truth')
axs[1].imshow(ground_truth)
plt.show()
```

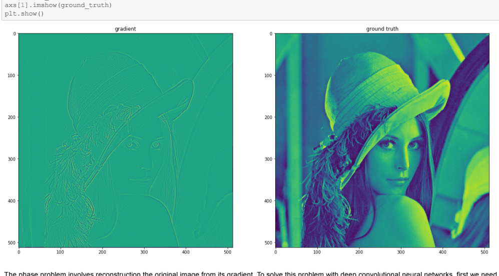

The phase problem involves reconstruction the original image from its gradient. To solve this problem with deep convolutional neural networks, first we need to check if the framework tensorflow has been installed or not. This can be done by

In [2]:
```
import tensorflow
print( tensorflow.__version__)
```

```
/usr/lib/python3.7/site-packages/tensorflow/python/framework/dtypes.py:526: FutureWarning: Passing (type, 1) or '1type' a
s a synonym of type is deprecated; in a future version of numpy, it will be understood as (type, (1,)) / '(1,)type'.
  _np_qint8 = np.dtype([("qint8", np.int8, 1)])
/usr/lib/python3.7/site-packages/tensorflow/python/framework/dtypes.py:527: FutureWarning: Passing (type, 1) or '1type' a
s a synonym of type is deprecated; in a future version of numpy, it will be understood as (type, (1,)) / '(1,)type'.
  _np_quint8 = np.dtype([("quint8", np.uint8, 1)])
/usr/lib/python3.7/site-packages/tensorflow/python/framework/dtypes.py:528: FutureWarning: Passing (type, 1) or '1type' a
s a synonym of type is deprecated; in a future version of numpy, it will be understood as (type, (1,)) / '(1,)type'.
  _np_qint16 = np.dtype([("qint16", np.int16, 1)])
/usr/lib/python3.7/site-packages/tensorflow/python/framework/dtypes.py:529: FutureWarning: Passing (type, 1) or '1type' a
s a synonym of type is deprecated; in a future version of numpy, it will be understood as (type, (1,)) / '(1,)type'.
  _np_quint16 = np.dtype([("quint16", np.uint16, 1)])
/usr/lib/python3.7/site-packages/tensorflow/python/framework/dtypes.py:530: FutureWarning: Passing (type, 1) or '1type' a
s a synonym of type is deprecated; in a future version of numpy, it will be understood as (type, (1,)) / '(1,)type'.
  _np_qint32 = np.dtype([("qint32", np.int32, 1)])
/usr/lib/python3.7/site-packages/tensorflow/python/framework/dtypes.py:535: FutureWarning: Passing (type, 1) or '1type' a
s a synonym of type is deprecated; in a future version of numpy, it will be understood as (type, (1,)) / '(1,)type'.
  np_resource = np.dtype([("resource", np.ubyte, 1)])
1.13.1
```

Then we start to implement our MCNN model. First we define our kernel initializer and regularizer

In [3]:
```
from tensorflow.python.keras import regularizers
from tensorflow.python.keras.initializers import he_normal
kr = regularizers.l2( 1.0e-3 )
ki = he_normal()
```

With `kr` and `ki`, we define the classic U-Net structure

In [4]:
```
from tensorflow.python.keras.layers import Input
from tensorflow.python.keras.layers import Conv2D, Conv2DTranspose
from tensorflow.python.keras.models import Model
from tensorflow.python.keras.layers.merge import add

init = Input(shape=(512, 512, 1))
init_ = Conv2D( 32, kernel_size=(31, 31), activation = "relu", strides = 1, padding="same", kernel_regularizer=kr, kernel_ini
tializer=ki )( init )
l1 = Conv2D( 64, kernel_size=(7, 7), activation = "relu", strides = 2, padding="same", kernel_regularizer=kr, kernel_initiali
zer=ki )( init_ )
l2=Conv2D(128, kernel_size=(5, 5), activation="relu", strides=2, padding='same', kernel_regularizer=kr, kernel_initializer=ki
)(l1) # 128
l3=Conv2D(192, kernel_size=(3, 3), activation="relu", strides=2, padding='same', kernel_regularizer=kr, kernel_initializer=ki
)(l2) # 64
l4=Conv2D(256, kernel_size=(3, 3), activation="relu", strides=2, padding='same', kernel_regularizer=kr, kernel_initializer=ki
)(l3) # 32
l5=Conv2D(384, kernel_size=(3, 3), activation="relu", strides=2, padding='same', kernel_regularizer=kr, kernel_initializer=ki
)(l4) # 16
l6=Conv2D(512, kernel_size=(3, 3), activation="relu", strides=2, padding='same', kernel_regularizer=kr, kernel_initializer=ki
)(l5) # 8
lx1=Conv2D(768, kernel_size=(3, 3), activation="relu", strides=(1,1), padding='valid', kernel_regularizer=kr, kernel_initiali
zer=ki)(l6) # 6
lx1=Conv2D(1024, kernel_size=(3, 3), activation="relu", strides=(1,1), padding='valid', kernel_regularizer=kr, kernel_initial
izer=ki)(lx1) # 4
lx2=Conv2D(2048, kernel_size=(3, 3), activation="relu", strides=(1,1), padding='valid', kernel_regularizer=kr, kernel_initial
izer=ki)(lx1) # 2
lx3=Conv2DTranspose(1024, kernel_size=(3, 3), strides=(1,1), padding='valid', kernel_regularizer=kr, kernel_initializer=ki, a
ctivation="relu")(lx2) # 4
lx4=add([lx3, lx1])
lx5=Conv2DTranspose(768, kernel_size=(3, 3), strides=(1,1), padding='valid', kernel_regularizer=kr, kernel_initializer=ki, ac
tivation="relu")(lx4) # 6
l7=Conv2DTranspose(512, kernel_size=(3, 3), strides=(1,1), padding='valid', kernel_regularizer=kr, kernel_initializer=ki, act
ivation="relu")(lx5) # 8
l9=add([l7, l6]) #
l10=Conv2DTranspose(384, kernel_size=(3, 3), strides=(2,2), padding='same', kernel_regularizer=kr, kernel_initializer=ki, act
ivation="relu")(l9) # 16
l12=add([l10, l5]) #
l13=Conv2DTranspose(256, kernel_size=(3, 3),  activation="relu", strides=(2,2), padding='same', kernel_regularizer=kr, kernel
_initializer=ki)(l12) # 32
l15=add([l13, l4]) #
l16=Conv2DTranspose(192, kernel_size=(3, 3), strides=(2,2), activation="relu", padding='same', kernel_regularizer=kr, kernel_
initializer=ki)(l15) # 64
l18=add([l16, l3]) #
l19=Conv2DTranspose(128, kernel_size=(3, 3), activation="relu", strides=(2,2), padding='same', kernel_regularizer=kr, kernel_
initializer=ki)(l18) # 128
l21=add([l19, l2]) #
l22=Conv2DTranspose(64, kernel_size=(3, 3), activation="relu", strides=(2,2), padding='same', kernel_regularizer=kr, kernel_i
nitializer=ki)(l21) # 256
l24=add([l22, l1])
l25=Conv2DTranspose(32, kernel_size=(3, 3), activation="relu", strides=(2,2), padding='same', kernel_regularizer=kr, kernel_i
nitializer=ki)(l24) # 512
l27=add([l25, init_])
llast=Conv2D(16 , kernel_size=(3, 3), activation='relu', strides=(1,1), padding='same', kernel_regularizer=kr, kernel_initial
izer=ki)(l27)
llast=Conv2D(8 , kernel_size=(3, 3), activation='relu', strides=(1,1), padding='same', kernel_regularizer=kr, kernel_initiali
zer=ki)(llast)
last512=Conv2D(1, kernel_size=(31, 31), activation='sigmoid', strides=(1,1), padding='same', kernel_regularizer=kr, kernel_in
itializer=ki, name="gen_512")(llast)
#unet = Model( init, last512 )
```

```
WARNING:tensorflow:From /usr/lib/python3.7/site-packages/tensorflow/python/ops/resource_variable_ops.py:435: colocate_wit
h (from tensorflow.python.framework.ops) is deprecated and will be removed in a future version.
Instructions for updating:
Colocations handled automatically by placer.
```

MCNN expands the layers in the decoder to match low-frequency components. The expanded layers can be defined this way to make a model

In [5]:
```
last4=Conv2D(8 , kernel_size=(3, 3), activation='relu', strides=(1,1), padding='same', kernel_regularizer=kr, kernel_initiali
zer=ki)(lx4)
last4=Conv2D(1, kernel_size=(3, 3), activation='sigmoid', strides=(1,1), padding='same', kernel_regularizer=kr, kernel_initia
lizer=ki, name="gen_4")(last4)

last8=Conv2D(8 , kernel_size=(3, 3), activation='relu', strides=(1,1), padding='same', kernel_regularizer=kr, kernel_initiali
zer=ki)(l9)
last8=Conv2D(1, kernel_size=(5, 5), activation='sigmoid', strides=(1,1), padding='same', kernel_regularizer=kr, kernel_initia
lizer=ki, name="gen_8")(last8)

last16=Conv2D(8 , kernel_size=(3, 3), activation='relu', strides=(1,1), padding='same', kernel_regularizer=kr, kernel_initial
izer=ki)(l12)
last16=Conv2D(1, kernel_size=(5, 5), activation='sigmoid', strides=(1,1), padding='same', kernel_regularizer=kr, kernel_initi
alizer=ki, name="gen_16")(last16)

last32=Conv2D(8 , kernel_size=(3, 3), activation='relu', strides=(1,1), padding='same', kernel_regularizer=kr, kernel_initial
izer=ki)(l15)
last32=Conv2D(1, kernel_size=(7, 7), activation='sigmoid', strides=(1,1), padding='same', kernel_regularizer=kr, kernel_initi
alizer=ki, name="gen_32")(last32)

last64=Conv2D(8 , kernel_size=(3, 3), activation='relu', strides=(1,1), padding='same', kernel_regularizer=kr, kernel_initial
izer=ki)(l18)
last64=Conv2D(1, kernel_size=(9, 9), activation='sigmoid', strides=(1,1), padding='same', kernel_regularizer=kr, kernel_initi
alizer=ki, name="gen_64")(last64)

last128=Conv2D(8 , kernel_size=(3, 3), activation='relu', strides=(1,1), padding='same', kernel_regularizer=kr, kernel_initia
lizer=ki)(l21)
last128=Conv2D(1, kernel_size=(11, 11), activation='sigmoid', strides=(1,1), padding='same', kernel_regularizer=kr, kernel_in
itializer=ki, name="gen_128")(last128)

last256=Conv2D(8 , kernel_size=(3, 3), activation='relu', strides=(1,1), padding='same', kernel_regularizer=kr, kernel_initia
lizer=ki)(l24)
last256=Conv2D(1, kernel_size=(17, 17), activation='sigmoid', strides=(1,1), padding='same', kernel_regularizer=kr, kernel_in
itializer=ki, name="gen_256")(last256)

mcnn = Model(inputs=init, outputs=[last512, last256, last128, last64, last32, last16, last8, last4])
mcnn.summary()
```

Layer (type)	Output Shape	Param #	Connected to
input_1 (InputLayer)	(None, 512, 512, 1)	0	
conv2d (Conv2D)	(None, 512, 512, 32)	30784	input_1[0][0]
conv2d_1 (Conv2D)	(None, 256, 256, 64)	100416	conv2d[0][0]
conv2d_2 (Conv2D)	(None, 128, 128, 128	204928	conv2d_1[0][0]
conv2d_3 (Conv2D)	(None, 64, 64, 192)	221376	conv2d_2[0][0]
conv2d_4 (Conv2D)	(None, 32, 32, 256)	442624	conv2d_3[0][0]
conv2d_5 (Conv2D)	(None, 16, 16, 384)	885120	conv2d_4[0][0]
conv2d_6 (Conv2D)	(None, 8, 8, 512)	1769984	conv2d_5[0][0]
conv2d_7 (Conv2D)	(None, 6, 6, 768)	3539712	conv2d_6[0][0]
conv2d_8 (Conv2D)	(None, 4, 4, 1024)	7078912	conv2d_7[0][0]
conv2d_9 (Conv2D)	(None, 2, 2, 2048)	18876416	conv2d_8[0][0]
conv2d_transpose (Conv2DTranspo	(None, 4, 4, 1024)	18875392	conv2d_9[0][0]
add (Add)	(None, 4, 4, 1024)	0	conv2d_transpose[0][0]<br>conv2d_8[0][0]
conv2d_transpose_1 (Conv2DTrans	(None, 6, 6, 768)	7078656	add[0][0]
conv2d_transpose_2 (Conv2DTrans	(None, 8, 8, 512)	3539456	conv2d_transpose_1[0][0]
add_1 (Add)	(None, 8, 8, 512)	0	conv2d_transpose_2[0][0]<br>conv2d_6[0][0]
conv2d_transpose_3 (Conv2DTrans	(None, 16, 16, 384)	1769856	add_1[0][0]
add_2 (Add)	(None, 16, 16, 384)	0	conv2d_transpose_3[0][0]<br>conv2d_5[0][0]
conv2d_transpose_4 (Conv2DTrans	(None, 32, 32, 256)	884992	add_2[0][0]
add_3 (Add)	(None, 32, 32, 256)	0	conv2d_transpose_4[0][0]<br>conv2d_4[0][0]
conv2d_transpose_5 (Conv2DTrans	(None, 64, 64, 192)	442560	add_3[0][0]
add_4 (Add)	(None, 64, 64, 192)	0	conv2d_transpose_5[0][0]<br>conv2d_3[0][0]
conv2d_transpose_6 (Conv2DTrans	(None, 128, 128, 128	221312	add_4[0][0]
add_5 (Add)	(None, 128, 128, 128	0	conv2d_transpose_6[0][0]<br>conv2d_2[0][0]
conv2d_transpose_7 (Conv2DTrans	(None, 256, 256, 64)	73792	add_5[0][0]
add_6 (Add)	(None, 256, 256, 64)	0	conv2d_transpose_7[0][0]<br>conv2d_1[0][0]
conv2d_transpose_8 (Conv2DTrans	(None, 512, 512, 32)	18464	add_6[0][0]
add_7 (Add)	(None, 512, 512, 32)	0	conv2d_transpose_8[0][0]<br>conv2d[0][0]
conv2d_10 (Conv2D)	(None, 512, 512, 16)	4624	add_7[0][0]
conv2d_11 (Conv2D)	(None, 512, 512, 8)	1160	conv2d_10[0][0]
conv2d_18 (Conv2D)	(None, 256, 256, 8)	4616	add_6[0][0]
conv2d_17 (Conv2D)	(None, 128, 128, 8)	9224	add_5[0][0]
conv2d_16 (Conv2D)	(None, 64, 64, 8)	13832	add_4[0][0]
conv2d_15 (Conv2D)	(None, 32, 32, 8)	18440	add_3[0][0]
conv2d_14 (Conv2D)	(None, 16, 16, 8)	27656	add_2[0][0]
conv2d_13 (Conv2D)	(None, 8, 8, 8)	36872	add_1[0][0]
conv2d_12 (Conv2D)	(None, 4, 4, 8)	73736	add[0][0]
gen_512 (Conv2D)	(None, 512, 512, 1)	7689	conv2d_11[0][0]
gen_256 (Conv2D)	(None, 256, 256, 1)	2313	conv2d_18[0][0]
gen_128 (Conv2D)	(None, 128, 128, 1)	969	conv2d_17[0][0]
gen_64 (Conv2D)	(None, 64, 64, 1)	649	conv2d_16[0][0]
gen_32 (Conv2D)	(None, 32, 32, 1)	393	conv2d_15[0][0]
gen_16 (Conv2D)	(None, 16, 16, 1)	201	conv2d_14[0][0]
gen_8 (Conv2D)	(None, 8, 8, 1)	201	conv2d_13[0][0]
gen_4 (Conv2D)	(None, 4, 4, 1)	73	conv2d_12[0][0]

```
Total params: 66,257,400
Trainable params: 66,257,400
Non-trainable params: 0
```

With 2 GPUs, we make the training plan using Adam minimizing the mean absolute errors

In [6]:
```
from tensorflow.python.keras.utils import multi_gpu_model
mcnn = multi_gpu_model( mcnn, gpus=2 )
mcnn.compile( loss='mae', optimizer='adam' )
```

The low frequency components data can be generated by pooling the original images sequencially. This is done by

In [7]:
```
from skimage.measure import block_reduce
def make_block_reduce( input_layers, dim=(2,2), mode=np.mean ):
    stacked_layers = [ block_reduce( image, dim, mode ) for image in input_layers ]
    return np.asarray( stacked_layers, dtype='float32' )
```

Then by simulating the gradient images from some existing image set, for example ImageNet, we can prepare a training/validation dataset with 1024 samples this way

In [8]:
```
import glob
paths = glob.glob('/raid/feng/pictures/openimagenet.v5/*.jpg')

import random
random.shuffle( paths )

shape = (512, 512)
number = 1024
gaussian_noise_var = 0.01

from imageio import imread
def trim_image_from_path( image_path, shape ):
    try:
        image = imread( image_path )
    except:
        return None
    image = np.asarray( image, dtype='float32' )
    image = image / 255.0

    if 3 == len(image.shape): # rgb -> gray
        image = 0.2627*image[:,:,0]+0.6780*image[:,:,1]+0.0593*image[:,:,2]

    scaling_ratio = [ int(a/b) for a, b in zip( image.shape, shape ) ]
    if scaling_ratio[0] > 1 and scaling_ratio[1] > 1 :
        image = block_reduce( image, tuple(scaling_ratio), np.mean )

    dim_diff = [ (a - b)>>1 for a, b in zip( image.shape, shape ) ]
    if dim_diff[0] < 0 or dim_diff[1] < 0:
        return None
    return image[dim_diff[0]:dim_diff[0]+shape[0], dim_diff[1]:dim_diff[1]+shape[1]]

output_images = []
input_images = []
counter = 0
for path in paths:
    trimmed_image = trim_image_from_path(path, shape)
    if trimmed_image is not None:
        print( f'preparing training set from {path} - {counter}/{number}', end='\r' )
        output_images.append(trimmed_image)
        input_images.append( convolve( trimmed_image, kernel, mode='same' ) )
        counter += 1
    if counter == number:
        break

input_layers = np.asarray( input_images, dtype='float32' ).reshape( (number,)+shape+(1,) )
input_layers += np.random.normal(loc=0.0, scale=gaussian_noise_var, size=input_layers.shape)

output_layers = [np.asarray( output_images, dtype='float32' ), None, None, None, None, None, None, None]
for idx in range( 7 ):
    output_layers[idx+1] = make_block_reduce( output_layers[idx], (2,2), np.mean )
    output_layers[idx] = output_layers[idx].reshape( output_layers[idx].shape + (1,) )
output_layers[7] = output_layers[7].reshape( output_layers[7].shape + (1,) )
```

```
preparing training set from /raid/feng/pictures/openimagenet.v5/14941980752_2596843ff0_o.jpg - 29/1024
/usr/lib/python3.7/site-packages/skimage/util/shape.py:93: RuntimeWarning: Cannot provide views on a non-contiguous input
array without copying.
  warn(RuntimeWarning("Cannot provide views on a non-contiguous input "
preparing training set from /raid/feng/pictures/openimagenet.v5/15680766571_07c8d79f35_o.jpg - 792/1024
/usr/lib/python3.7/site-packages/PIL/TiffImagePlugin.py:784: UserWarning: Possibly corrupt EXIF data.  Expecting to read
18481152 bytes but only got 0. Skipping tag 0
  " Skipping tag %s" % (size, len(data), tag)
/usr/lib/python3.7/site-packages/PIL/TiffImagePlugin.py:784: UserWarning: Possibly corrupt EXIF data.  Expecting to read
33619968 bytes but only got 0. Skipping tag 0
  " Skipping tag %s" % (size, len(data), tag)
/usr/lib/python3.7/site-packages/PIL/TiffImagePlugin.py:784: UserWarning: Possibly corrupt EXIF data.  Expecting to read
4718592 bytes but only got 0. Skipping tag 0
  " Skipping tag %s" % (size, len(data), tag)
/usr/lib/python3.7/site-packages/PIL/TiffImagePlugin.py:784: UserWarning: Possibly corrupt EXIF data.  Expecting to read
31197188 bytes but only got 0. Skipping tag 0
  " Skipping tag %s" % (size, len(data), tag)
/usr/lib/python3.7/site-packages/PIL/TiffImagePlugin.py:802: UserWarning: Corrupt EXIF data.  Expecting to read 12 bytes
but only got 0.
  warnings.warn(str(msg))
preparing training set from /raid/feng/pictures/openimagenet.v5/14769660060_c15f2cb9b0_o.jpg - 1023/1024
```

The gradient and the ground truth for the first image-pair are visualized below

In [9]:
```
fig, axs = plt.subplots(1, 2)
axs[0].set_title('gradient - simulated')
axs[0].imshow(np.squeeze(input_layers[0]))
axs[1].set_title('ground truth')
axs[1].imshow(np.squeeze(output_layers[0][0]))
plt.rcParams["figure.figsize"] = (20,10)
plt.show()
```

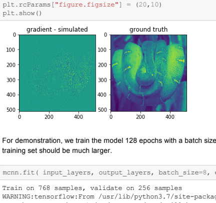

For demonstration, we train the model 128 epochs with a batch size of 8 and a validation split of 1/4. In actual applications, the epochs and the size of the training set should be much larger.

In [10]:
```
mcnn.fit( input_layers, output_layers, batch_size=8, epochs=128, verbose=1,validation_split=0.25 )
```

```
Train on 768 samples, validate on 256 samples
WARNING:tensorflow:From /usr/lib/python3.7/site-packages/tensorflow/python/ops/math_ops.py:3066: to_int32 (from tensorflo
w.python.ops.math_ops) is deprecated and will be removed in a future version.
Instructions for updating:
Use tf.cast instead.
[illegible]
Epoch 113/128
768/768 [==============================] - 64s 84ms/sample - loss: 2.6076 - gen_512_loss: 0.2924 - gen_256_loss: 0.3330 - gen_128_loss: 0.3006 - gen_64_loss: 0.2739 - gen_32_loss: 0.2534 - gen_16_loss: 0.2340 - gen_8_loss: 0.2065 - gen_4_loss: 0.1881 - val_loss: 4.3354 - val_gen_512_loss: 0.4810 - val_gen_256_loss: 0.5203 - val_gen_128_loss: 0.5006 - val_gen_64_loss: 0.4827 - val_gen_32_loss: 0.4683 - val_gen_16_loss: 0.4542 - val_gen_8_loss: 0.4384 - val_gen_4_loss: 0.4636
Epoch 114/128
768/768 [==============================] - 66s 86ms/sample - loss: 2.5378 - gen_512_loss: 0.2869 - gen_256_loss: 0.3238 - gen_128_loss: 0.2910 - gen_64_loss: 0.2643 - gen_32_loss: 0.2443 - gen_16_loss: 0.2248 - gen_8_loss: 0.1984 - gen_4_loss: 0.1792 - val_loss: 4.2986 - val_gen_512_loss: 0.4809 - val_gen_256_loss: 0.5128 - val_gen_128_loss: 0.4874 - val_gen_64_loss: 0.4727 - val_gen_32_loss: 0.4631 - val_gen_16_loss: 0.4583 - val_gen_8_loss: 0.4403 - val_gen_4_loss: 0.4585
Epoch 115/128
768/768 [==============================] - 67s 87ms/sample - loss: 2.4724 - gen_512_loss: 0.2728 - gen_256_loss: 0.3156 - gen_128_loss: 0.2832 - gen_64_loss: 0.2568 - gen_32_loss: 0.2376 - gen_16_loss: 0.2190 - gen_8_loss: 0.1918 - gen_4_loss: 0.1723 - val_loss: 4.2244 - val_gen_512_loss: 0.4763 - val_gen_256_loss: 0.5041 - val_gen_128_loss: 0.4811 - val_gen_64_loss: 0.4622 - val_gen_32_loss: 0.4533 - val_gen_16_loss: 0.4436 - val_gen_8_loss: 0.4295 - val_gen_4_loss: 0.4515
Epoch 116/128
768/768 [==============================] - 66s 86ms/sample - loss: 2.6311 - gen_512_loss: 0.2998 - gen_256_loss: 0.3369 - gen_128_loss: 0.3040 - gen_64_loss: 0.2770 - gen_32_loss: 0.2568 - gen_16_loss: 0.2351 - gen_8_loss: 0.2079 - gen_4_loss: 0.1886 - val_loss: 4.2840 - val_gen_512_loss: 0.4968 - val_gen_256_loss: 0.5155 - val_gen_128_loss: 0.4911 - val_gen_64_loss: 0.4732 - val_gen_32_loss: 0.4617 - val_gen_16_loss: 0.4480 - val_gen_8_loss: 0.4299 - val_gen_4_loss: 0.4437
Epoch 117/128
768/768 [==============================] - 65s 85ms/sample - loss: 2.6111 - gen_512_loss: 0.2952 - gen_256_loss: 0.3351 - gen_128_loss: 0.3016 - gen_64_loss: 0.2745 - gen_32_loss: 0.2542 - gen_16_loss: 0.2335 - gen_8_loss: 0.2066 - gen_4_loss: 0.1861 - val_loss: 4.3211 - val_gen_512_loss: 0.4842 - val_gen_256_loss: 0.5156 - val_gen_128_loss: 0.4934 - val_gen_64_loss: 0.4772 - val_gen_32_loss: 0.4667 - val_gen_16_loss: 0.4587 - val_gen_8_loss: 0.4377 - val_gen_4_loss: 0.4635
Epoch 118/128
768/768 [==============================] - 65s 85ms/sample - loss: 2.5957 - gen_512_loss: 0.3008 - gen_256_loss: 0.3302 - gen_128_loss: 0.2978 - gen_64_loss: 0.2714 - gen_32_loss: 0.2507 - gen_16_loss: 0.2311 - gen_8_loss: 0.2046 - gen_4_loss: 0.1842 - val_loss: 4.2792 - val_gen_512_loss: 0.4773 - val_gen_256_loss: 0.5143 - val_gen_128_loss: 0.4907 - val_gen_64_loss: 0.4731 - val_gen_32_loss: 0.4615 - val_gen_16_loss: 0.4543 - val_gen_8_loss: 0.4372 - val_gen_4_loss: 0.4456
Epoch 119/128
768/768 [==============================] - 65s 84ms/sample - loss: 2.5196 - gen_512_loss: 0.2827 - gen_256_loss: 0.3209 - gen_128_loss: 0.2895 - gen_64_loss: 0.2628 - gen_32_loss: 0.2433 - gen_16_loss: 0.2234 - gen_8_loss: 0.1969 - gen_4_loss: 0.1757 - val_loss: 4.4213 - val_gen_512_loss: 0.5012 - val_gen_256_loss: 0.5302 - val_gen_128_loss: 0.5083 - val_gen_64_loss: 0.4925 - val_gen_32_loss: 0.4804 - val_gen_16_loss: 0.4707 - val_gen_8_loss: 0.4512 - val_gen_4_loss: 0.4628
Epoch 120/128
768/768 [==============================] - 67s 87ms/sample - loss: 2.5269 - gen_512_loss: 0.2871 - gen_256_loss: 0.3213 - gen_128_loss: 0.2899 - gen_64_loss: 0.2632 - gen_32_loss: 0.2440 - gen_16_loss: 0.2240 - gen_8_loss: 0.1966 - gen_4_loss: 0.1769 - val_loss: 4.2571 - val_gen_512_loss: 0.4887 - val_gen_256_loss: 0.5086 - val_gen_128_loss: 0.4857 - val_gen_64_loss: 0.4679 - val_gen_32_loss: 0.4571 - val_gen_16_loss: 0.4474 - val_gen_8_loss: 0.4272 - val_gen_4_loss: 0.4508
Epoch 121/128
768/768 [==============================] - 64s 84ms/sample - loss: 2.5758 - gen_512_loss: 0.2892 - gen_256_loss: 0.3290 - gen_128_loss: 0.2958 - gen_64_loss: 0.2694 - gen_32_loss: 0.2500 - gen_16_loss: 0.2310 - gen_8_loss: 0.2028 - gen_4_loss: 0.1849 - val_loss: 4.2268 - val_gen_512_loss: 0.4722 - val_gen_256_loss: 0.5038 - val_gen_128_loss: 0.4809 - val_gen_64_loss: 0.4655 - val_gen_32_loss: 0.4559 - val_gen_16_loss: 0.4457 - val_gen_8_loss: 0.4285 - val_gen_4_loss: 0.4500
Epoch 122/128
768/768 [==============================] - 63s 82ms/sample - loss: 2.5298 - gen_512_loss: 0.2831 - gen_256_loss: 0.3215 - gen_128_loss: 0.2896 - gen_64_loss: 0.2639 - gen_32_loss: 0.2450 - gen_16_loss: 0.2258 - gen_8_loss: 0.1991 - gen_4_loss: 0.1783 - val_loss: 4.1915 - val_gen_512_loss: 0.4706 - val_gen_256_loss: 0.4974 - val_gen_128_loss: 0.4766 - val_gen_64_loss: 0.4614 - val_gen_32_loss: 0.4513 - val_gen_16_loss: 0.4415 - val_gen_8_loss: 0.4243 - val_gen_4_loss: 0.4454
Epoch 123/128
768/768 [==============================] - 67s 87ms/sample - loss: 2.5251 - gen_512_loss: 0.2823 - gen_256_loss: 0.3214 - gen_128_loss: 0.2898 - gen_64_loss: 0.2634 - gen_32_loss: 0.2445 - gen_16_loss: 0.2246 - gen_8_loss: 0.1974 - gen_4_loss: 0.1791 - val_loss: 4.2805 - val_gen_512_loss: 0.4790 - val_gen_256_loss: 0.5114 - val_gen_128_loss: 0.4902 - val_gen_64_loss: 0.4733 - val_gen_32_loss: 0.4608 - val_gen_16_loss: 0.4549 - val_gen_8_loss: 0.4349 - val_gen_4_loss: 0.4534
Epoch 124/128
768/768 [==============================] - 65s 84ms/sample - loss: 2.5901 - gen_512_loss: 0.2945 - gen_256_loss: 0.3297 - gen_128_loss: 0.2976 - gen_64_loss: 0.2722 - gen_32_loss: 0.2538 - gen_16_loss: 0.2320 - gen_8_loss: 0.2052 - gen_4_loss: 0.1821 - val_loss: 4.3285 - val_gen_512_loss: 0.4872 - val_gen_256_loss: 0.5154 - val_gen_128_loss: 0.4950 - val_gen_64_loss: 0.4789 - val_gen_32_loss: 0.4705 - val_gen_16_loss: 0.4593 - val_gen_8_loss: 0.4433 - val_gen_4_loss: 0.4558
Epoch 125/128
768/768 [==============================] - 67s 87ms/sample - loss: 2.7426 - gen_512_loss: 0.3178 - gen_256_loss: 0.3480 - gen_128_loss: 0.3159 - gen_64_loss: 0.2894 - gen_32_loss: 0.2710 - gen_16_loss: 0.2509 - gen_8_loss: 0.2225 - gen_4_loss: 0.2021 - val_loss: 4.4128 - val_gen_512_loss: 0.5235 - val_gen_256_loss: 0.5277 - val_gen_128_loss: 0.5001 - val_gen_64_loss: 0.4831 - val_gen_32_loss: 0.4748 - val_gen_16_loss: 0.4633 - val_gen_8_loss: 0.4469 - val_gen_4_loss: 0.4656
Epoch 126/128
768/768 [==============================] - 65s 84ms/sample - loss: 2.5688 - gen_512_loss: 0.2898 - gen_256_loss: 0.3242 - gen_128_loss: 0.2937 - gen_64_loss: 0.2684 - gen_32_loss: 0.2488 - gen_16_loss: 0.2299 - gen_8_loss: 0.2035 - gen_4_loss: 0.1840 - val_loss: 4.4575 - val_gen_512_loss: 0.5001 - val_gen_256_loss: 0.5267 - val_gen_128_loss: 0.5098 - val_gen_64_loss: 0.4935 - val_gen_32_loss: 0.4862 - val_gen_16_loss: 0.4776 - val_gen_8_loss: 0.4625 - val_gen_4_loss: 0.4759
Epoch 127/128
768/768 [==============================] - 65s 85ms/sample - loss: 2.5644 - gen_512_loss: 0.2857 - gen_256_loss: 0.3246 - gen_128_loss: 0.2927 - gen_64_loss: 0.2673 - gen_32_loss: 0.2488 - gen_16_loss: 0.2295 - gen_8_loss: 0.2049 - gen_4_loss: 0.1852 - val_loss: 4.4776 - val_gen_512_loss: 0.5333 - val_gen_256_loss: 0.5370 - val_gen_128_loss: 0.5160 - val_gen_64_loss: 0.4940 - val_gen_32_loss: 0.4869 - val_gen_16_loss: 0.4669 - val_gen_8_loss: 0.4489 - val_gen_4_loss: 0.4684
Epoch 128/128
768/768 [==============================] - 67s 87ms/sample - loss: 2.5497 - gen_512_loss: 0.2893 - gen_256_loss: 0.3230 - gen_128_loss: 0.2913 - gen_64_loss: 0.2653 - gen_32_loss: 0.2467 - gen_16_loss: 0.2266 - gen_8_loss: 0.2001 - gen_4_loss: 0.1814 - val_loss: 4.3095 - val_gen_512_loss: 0.5166 - val_gen_256_loss: 0.5166 - val_gen_128_loss: 0.4957 - val_gen_64_loss: 0.4764 - val_gen_32_loss: 0.4665 - val_gen_16_loss: 0.4580 - val_gen_8_loss: 0.4409 - val_gen_4_loss: 0.4518
```

Out[10]: `<tensorflow.python.keras.callbacks.History at 0x7fdfc4666390>`

The training will take quite a while depending on the hardware.

After training, we can make a prediction to test

In [11]:
```
prediction, *_ = mcnn.predict( gradient.reshape((1,)+gradient.shape+(1,)))
prediction = np.squeeze( prediction )
```

Now we can compare the ground truth and the prediction

In [12]:
```
from mpl_toolkits.axes_grid1 import make_axes_locatable
fig, axs = plt.subplots(1, 4)
axs[0].set_title('prediction')
axs[0].imshow(prediction)
axs[1].set_title('ground truth')
axs[1].imshow(ground_truth)
axs[2].set_title('gradient')
axs[2].imshow(gradient)
ground_truth = (ground_truth-np.amin(ground_truth))/(np.amax(ground_truth)-np.amin(ground_truth)+1.0e-10)
prediction = (prediction-np.amin(prediction))/(np.amax(prediction)-np.amin(prediction)+1.0e-10)
diff = ground_truth - prediction
axs[3].set_title('difference')
im = axs[3].imshow(diff)
divider = make_axes_locatable(axs[3])
cax = divider.append_axes('right', size='5%', pad=0.05)
fig.colorbar(im, cax=cax, orientation='vertical')
plt.rcParams["figure.figsize"] = (20,10)
plt.show()

print( f'MAE is {np.sum(np.abs(diff))/(512*512)}' )
```

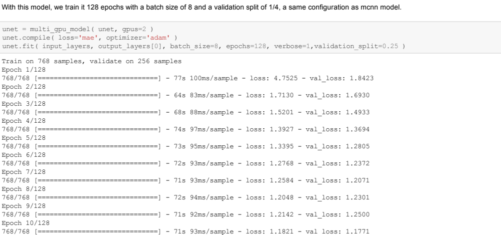


```
MAE is 0.06797673713645039
```

We can compare its performance with a conventional U-Net model.

In [13]:
```
init = Input(shape=(512, 512, 1))
init_ = Conv2D( 32, kernel_size=(31, 31), activation = "relu", strides = 1, padding="same", kernel_regularizer=kr, kernel_ini
tializer=ki )( init )
l1 = Conv2D( 64, kernel_size=(7, 7), activation = "relu", strides = 2, padding="same", kernel_regularizer=kr, kernel_initiali
zer=ki )( init_ )
l2=Conv2D(128, kernel_size=(5, 5), activation="relu", strides=2, padding='same', kernel_regularizer=kr, kernel_initializer=ki
)(l1) # 128
l3=Conv2D(192, kernel_size=(3, 3), activation="relu", strides=2, padding='same', kernel_regularizer=kr, kernel_initializer=ki
)(l2) # 64
l4=Conv2D(256, kernel_size=(3, 3), activation="relu", strides=2, padding='same', kernel_regularizer=kr, kernel_initializer=ki
)(l3) # 32
l5=Conv2D(384, kernel_size=(3, 3), activation="relu", strides=2, padding='same', kernel_regularizer=kr, kernel_initializer=ki
)(l4) # 16
l6=Conv2D(512, kernel_size=(3, 3), activation="relu", strides=2, padding='same', kernel_regularizer=kr, kernel_initializer=ki
)(l5) # 8
lx1=Conv2D(768, kernel_size=(3, 3), activation="relu", strides=(1,1), padding='valid', kernel_regularizer=kr, kernel_initiali
zer=ki)(l6) # 6
lx1=Conv2D(1024, kernel_size=(3, 3), activation="relu", strides=(1,1), padding='valid', kernel_regularizer=kr, kernel_initial
izer=ki)(lx1) # 4
lx2=Conv2D(2048, kernel_size=(3, 3), activation="relu", strides=(1,1), padding='valid', kernel_regularizer=kr, kernel_initial
izer=ki)(lx1) # 2
lx3=Conv2DTranspose(1024, kernel_size=(3, 3), strides=(1,1), padding='valid', kernel_regularizer=kr, kernel_initializer=ki, a
ctivation="relu")(lx2) # 4
lx4=add([lx3, lx1])
lx5=Conv2DTranspose(768, kernel_size=(3, 3), strides=(1,1), padding='valid', kernel_regularizer=kr, kernel_initializer=ki, ac
tivation="relu")(lx4) # 6
l7=Conv2DTranspose(512, kernel_size=(3, 3), strides=(1,1), padding='valid', kernel_regularizer=kr, kernel_initializer=ki, act
ivation="relu")(lx5) # 8
l9=add([l7, l6]) #
l10=Conv2DTranspose(384, kernel_size=(3, 3), strides=(2,2), padding='same', kernel_regularizer=kr, kernel_initializer=ki, act
ivation="relu")(l9) # 16
l12=add([l10, l5]) #
l13=Conv2DTranspose(256, kernel_size=(3, 3),  activation="relu", strides=(2,2), padding='same', kernel_regularizer=kr, kernel
_initializer=ki)(l12) # 32
l15=add([l13, l4]) #
l16=Conv2DTranspose(192, kernel_size=(3, 3), strides=(2,2), activation="relu", padding='same', kernel_regularizer=kr, kernel_
initializer=ki)(l15) # 64
l18=add([l16, l3]) #
l19=Conv2DTranspose(128, kernel_size=(3, 3), activation="relu", strides=(2,2), padding='same', kernel_regularizer=kr, kernel_
initializer=ki)(l18) # 128
l21=add([l19, l2]) #
l22=Conv2DTranspose(64, kernel_size=(3, 3), activation="relu", strides=(2,2), padding='same', kernel_regularizer=kr, kernel_i
nitializer=ki)(l21) # 256
l24=add([l22, l1])
l25=Conv2DTranspose(32, kernel_size=(3, 3), activation="relu", strides=(2,2), padding='same', kernel_regularizer=kr, kernel_i
nitializer=ki)(l24) # 512
l27=add([l25, init_])
llast=Conv2D(16 , kernel_size=(3, 3), activation='relu', strides=(1,1), padding='same', kernel_regularizer=kr, kernel_initial
izer=ki)(l27)
llast=Conv2D(8 , kernel_size=(3, 3), activation='relu', strides=(1,1), padding='same', kernel_regularizer=kr, kernel_initiali
zer=ki)(llast)
last512=Conv2D(1, kernel_size=(31, 31), activation='sigmoid', strides=(1,1), padding='same', kernel_regularizer=kr, kernel_in
itializer=ki, name="gen_512")(llast)
unet = Model( init, last512 )
```

With this model, we train it 128 epochs with a batch size of 8 and a validation split of 1/4, a same configuration as mcnn model.

In [16]:
```
unet = multi_gpu_model( unet, gpus=2 )
unet.compile( loss='mae', optimizer='adam' )
unet.fit( input_layers, output_layers[0], batch_size=8, epochs=128, verbose=1,validation_split=0.25 )
```

```
Train on 768 samples, validate on 256 samples
Epoch 1/128
768/768 [==============================] - 77s 100ms/sample - loss: 4.7525 - val_loss: 1.8423
Epoch 2/128
768/768 [==============================] - 64s 83ms/sample - loss: 1.7130 - val_loss: 1.6930
Epoch 3/128
768/768 [==============================] - 68s 88ms/sample - loss: 1.5201 - val_loss: 1.4933
Epoch 4/128
768/768 [==============================] - 74s 97ms/sample - loss: 1.3927 - val_loss: 1.3694
Epoch 5/128
768/768 [==============================] - 73s 95ms/sample - loss: 1.3395 - val_loss: 1.2805
Epoch 6/128
768/768 [==============================] - 72s 93ms/sample - loss: 1.2768 - val_loss: 1.2372
Epoch 7/128
768/768 [==============================] - 71s 93ms/sample - loss: 1.2584 - val_loss: 1.2071
Epoch 8/128
768/768 [==============================] - 72s 94ms/sample - loss: 1.2048 - val_loss: 1.2301
Epoch 9/128
768/768 [==============================] - 71s 92ms/sample - loss: 1.2142 - val_loss: 1.2500
Epoch 10/128
768/768 [==============================] - 71s 93ms/sample - loss: 1.1821 - val_loss: 1.1771
Epoch 11/128
768/768 [==============================] - 69s 90ms/sample - loss: 1.1707 - val_loss: 1.1435
Epoch 12/128
768/768 [==============================] - 71s 92ms/sample - loss: 1.1561 - val_loss: 1.1492
Epoch 13/128
768/768 [==============================] - 69s 89ms/sample - loss: 1.1333 - val_loss: 1.1203
Epoch 14/128
768/768 [==============================] - 71s 92ms/sample - loss: 1.1476 - val_loss: 1.1178
Epoch 15/128
768/768 [==============================] - 68s 89ms/sample - loss: 1.1166 - val_loss: 1.1397
Epoch 16/128
768/768 [==============================] - 71s 92ms/sample - loss: 1.1017 - val_loss: 1.1190
Epoch 17/128
768/768 [==============================] - 69s 90ms/sample - loss: 1.1159 - val_loss: 1.1660
Epoch 18/128
768/768 [==============================] - 69s 89ms/sample - loss: 1.1132 - val_loss: 1.2517
Epoch 19/128
768/768 [==============================] - 71s 92ms/sample - loss: 1.1026 - val_loss: 1.1669
Epoch 20/128
768/768 [==============================] - 68s 88ms/sample - loss: 1.0981 - val_loss: 1.0817
Epoch 21/128
768/768 [==============================] - 70s 91ms/sample - loss: 1.0756 - val_loss: 1.0845
Epoch 22/128
768/768 [==============================] - 72s 94ms/sample - loss: 1.0909 - val_loss: 1.2094
Epoch 23/128
768/768 [==============================] - 71s 93ms/sample - loss: 1.0770 - val_loss: 1.0819
Epoch 24/128
768/768 [==============================] - 70s 91ms/sample - loss: 1.0737 - val_loss: 1.1010
Epoch 25/128
768/768 [==============================] - 69s 90ms/sample - loss: 1.0691 - val_loss: 1.1204
Epoch 26/128
768/768 [==============================] - 72s 94ms/sample - loss: 1.0543 - val_loss: 1.1182
Epoch 27/128
768/768 [==============================] - 70s 91ms/sample - loss: 1.0644 - val_loss: 1.0613
Epoch 28/128
768/768 [==============================] - 68s 88ms/sample - loss: 1.0711 - val_loss: 1.0673
Epoch 29/128
768/768 [==============================] - 70s 91ms/sample - loss: 1.0544 - val_loss: 1.0978
Epoch 30/128
768/768 [==============================] - 70s 92ms/sample - loss: 1.0616 - val_loss: 1.0860
Epoch 31/128
768/768 [==============================] - 70s 91ms/sample - loss: 1.0387 - val_loss: 1.0662
Epoch 32/128
768/768 [==============================] - 71s 92ms/sample - loss: 1.0480 - val_loss: 1.0644
Epoch 33/128
768/768 [==============================] - 68s 89ms/sample - loss: 1.0621 - val_loss: 1.0700
Epoch 34/128
768/768 [==============================] - 68s 88ms/sample - loss: 1.0481 - val_loss: 1.0701
Epoch 35/128
768/768 [==============================] - 69s 89ms/sample - loss: 1.0362 - val_loss: 1.0571
Epoch 36/128
768/768 [==============================] - 69s 90ms/sample - loss: 1.0307 - val_loss: 1.0960
Epoch 37/128
768/768 [==============================] - 71s 92ms/sample - loss: 1.0461 - val_loss: 1.0730
Epoch 38/128
768/768 [==============================] - 68s 88ms/sample - loss: 1.0452 - val_loss: 1.0384
Epoch 39/128
768/768 [==============================] - 70s 92ms/sample - loss: 1.0178 - val_loss: 1.0508
Epoch 40/128
768/768 [==============================] - 69s 90ms/sample - loss: 1.0265 - val_loss: 1.0476
Epoch 41/128
768/768 [==============================] - 72s 94ms/sample - loss: 1.0283 - val_loss: 1.0481
Epoch 42/128
768/768 [==============================] - 70s 91ms/sample - loss: 1.0361 - val_loss: 1.0395
Epoch 43/128
768/768 [==============================] - 69s 90ms/sample - loss: 1.0265 - val_loss: 1.0701
Epoch 44/128
768/768 [==============================] - 70s 92ms/sample - loss: 1.0262 - val_loss: 1.0471
Epoch 45/128
768/768 [==============================] - 70s 91ms/sample - loss: 1.0365 - val_loss: 1.0559
Epoch 46/128
768/768 [==============================] - 70s 91ms/sample - loss: 1.0253 - val_loss: 1.0526
Epoch 47/128
768/768 [==============================] - 69s 90ms/sample - loss: 1.0213 - val_loss: 1.0459
Epoch 48/128
768/768 [==============================] - 70s 92ms/sample - loss: 1.0138 - val_loss: 1.0490
Epoch 49/128
768/768 [==============================] - 71s 92ms/sample - loss: 1.0048 - val_loss: 1.0299
Epoch 50/128
768/768 [==============================] - 69s 90ms/sample - loss: 1.0218 - val_loss: 1.0402
Epoch 51/128
768/768 [==============================] - 70s 91ms/sample - loss: 1.0305 - val_loss: 1.0265
Epoch 52/128
768/768 [==============================] - 69s 90ms/sample - loss: 0.9953 - val_loss: 1.0738
Epoch 53/128
768/768 [==============================] - 69s 90ms/sample - loss: 1.0135 - val_loss: 1.0396
Epoch 54/128
768/768 [==============================] - 69s 90ms/sample - loss: 1.0027 - val_loss: 1.0453
Epoch 55/128
768/768 [==============================] - 70s 91ms/sample - loss: 1.0150 - val_loss: 1.0396
Epoch 56/128
768/768 [==============================] - 73s 95ms/sample - loss: 1.0151 - val_loss: 1.0610
Epoch 57/128
768/768 [==============================] - 69s 90ms/sample - loss: 0.9958 - val_loss: 1.0324
Epoch 58/128
768/768 [==============================] - 68s 88ms/sample - loss: 1.0023 - val_loss: 1.0575
Epoch 59/128
768/768 [==============================] - 71s 93ms/sample - loss: 0.9905 - val_loss: 1.0199
Epoch 60/128
768/768 [==============================] - 70s 91ms/sample - loss: 1.0145 - val_loss: 1.0498
Epoch 61/128
768/768 [==============================] - 68s 89ms/sample - loss: 1.0086 - val_loss: 1.0369
Epoch 62/128
768/768 [==============================] - 68s 89ms/sample - loss: 1.0036 - val_loss: 1.0812
Epoch 63/128
768/768 [==============================] - 70s 91ms/sample - loss: 1.0037 - val_loss: 1.0847
Epoch 64/128
768/768 [==============================] - 69s 89ms/sample - loss: 0.9917 - val_loss: 1.0465
Epoch 65/128
768/768 [==============================] - 70s 91ms/sample - loss: 0.9918 - val_loss: 1.0682
Epoch 66/128
768/768 [==============================] - 69s 90ms/sample - loss: 1.0040 - val_loss: 1.0979
Epoch 67/128
768/768 [==============================] - 70s 91ms/sample - loss: 0.9875 - val_loss: 1.0570
Epoch 68/128
768/768 [==============================] - 68s 88ms/sample - loss: 0.9918 - val_loss: 1.0355
Epoch 69/128
768/768 [==============================] - 69s 90ms/sample - loss: 0.9941 - val_loss: 1.0366
Epoch 70/128
768/768 [==============================] - 71s 92ms/sample - loss: 0.9874 - val_loss: 1.1128
Epoch 71/128
768/768 [==============================] - 69s 90ms/sample - loss: 0.9866 - val_loss: 1.0621
Epoch 72/128
768/768 [==============================] - 72s 94ms/sample - loss: 0.9995 - val_loss: 1.1220
Epoch 73/128
768/768 [==============================] - 70s 91ms/sample - loss: 0.9899 - val_loss: 1.0479
Epoch 74/128
768/768 [==============================] - 70s 91ms/sample - loss: 0.9960 - val_loss: 1.0267
Epoch 75/128
768/768 [==============================] - 69s 90ms/sample - loss: 1.0000 - val_loss: 1.1329
Epoch 76/128
768/768 [==============================] - 70s 91ms/sample - loss: 0.9894 - val_loss: 1.0150
Epoch 77/128
768/768 [==============================] - 71s 92ms/sample - loss: 0.9939 - val_loss: 1.0731
Epoch 78/128
768/768 [==============================] - 69s 90ms/sample - loss: 1.0003 - val_loss: 1.0352
Epoch 79/128
768/768 [==============================] - 68s 89ms/sample - loss: 0.9836 - val_loss: 1.0829
Epoch 80/128
768/768 [==============================] - 68s 88ms/sample - loss: 0.9910 - val_loss: 1.0338
Epoch 81/128
768/768 [==============================] - 70s 92ms/sample - loss: 1.0015 - val_loss: 1.0392
Epoch 82/128
768/768 [==============================] - 71s 92ms/sample - loss: 0.9920 - val_loss: 1.0312
Epoch 83/128
768/768 [==============================] - 70s 91ms/sample - loss: 0.9937 - val_loss: 1.0159
Epoch 84/128
768/768 [==============================] - 70s 92ms/sample - loss: 0.9807 - val_loss: 1.0367
Epoch 85/128
768/768 [==============================] - 69s 90ms/sample - loss: 0.9819 - val_loss: 1.0377
Epoch 86/128
768/768 [==============================] - 71s 93ms/sample - loss: 0.9745 - val_loss: 1.0248
Epoch 87/128
768/768 [==============================] - 71s 92ms/sample - loss: 0.9805 - val_loss: 1.0179
Epoch 88/128
768/768 [==============================] - 70s 91ms/sample - loss: 0.9970 - val_loss: 1.0704
Epoch 89/128
768/768 [==============================] - 69s 89ms/sample - loss: 0.9814 - val_loss: 1.0397
Epoch 90/128
768/768 [==============================] - 68s 88ms/sample - loss: 0.9638 - val_loss: 1.0166
Epoch 91/128
768/768 [==============================] - 70s 91ms/sample - loss: 0.9711 - val_loss: 1.0384
Epoch 92/128
768/768 [==============================] - 69s 89ms/sample - loss: 0.9747 - val_loss: 1.0118
Epoch 93/128
768/768 [==============================] - 69s 90ms/sample - loss: 0.9912 - val_loss: 1.0282
Epoch 94/128
768/768 [==============================] - 71s 92ms/sample - loss: 0.9792 - val_loss: 1.0172
Epoch 95/128
768/768 [==============================] - 71s 92ms/sample - loss: 0.9621 - val_loss: 1.0184
Epoch 96/128
768/768 [==============================] - 70s 91ms/sample - loss: 0.9717 - val_loss: 1.0428
Epoch 97/128
768/768 [==============================] - 68s 89ms/sample - loss: 0.9666 - val_loss: 1.0541
Epoch 98/128
768/768 [==============================] - 70s 91ms/sample - loss: 0.9689 - val_loss: 1.0281
Epoch 99/128
768/768 [==============================] - 70s 91ms/sample - loss: 0.9905 - val_loss: 1.0427
Epoch 100/128
768/768 [==============================] - 69s 90ms/sample - loss: 1.0121 - val_loss: 1.0319
Epoch 101/128
768/768 [==============================] - 69s 90ms/sample - loss: 0.9726 - val_loss: 1.0542
Epoch 102/128
768/768 [==============================] - 70s 91ms/sample - loss: 0.9829 - val_loss: 1.0247
Epoch 103/128
768/768 [==============================] - 70s 91ms/sample - loss: 0.9774 - val_loss: 1.0515
Epoch 104/128
768/768 [==============================] - 68s 88ms/sample - loss: 0.9880 - val_loss: 1.0325
Epoch 105/128
768/768 [==============================] - 72s 93ms/sample - loss: 0.9715 - val_loss: 1.0104
Epoch 106/128
768/768 [==============================] - 69s 90ms/sample - loss: 0.9689 - val_loss: 1.0273
Epoch 107/128
768/768 [==============================] - 72s 94ms/sample - loss: 0.9803 - val_loss: 1.0318
Epoch 108/128
768/768 [==============================] - 69s 89ms/sample - loss: 0.9698 - val_loss: 1.0170
Epoch 109/128
768/768 [==============================] - 70s 91ms/sample - loss: 0.9572 - val_loss: 1.0580
Epoch 110/128
768/768 [==============================] - 71s 92ms/sample - loss: 0.9954 - val_loss: 1.0235
Epoch 111/128
768/768 [==============================] - 69s 90ms/sample - loss: 0.9685 - val_loss: 1.0119
Epoch 112/128
768/768 [==============================] - 68s 89ms/sample - loss: 0.9696 - val_loss: 1.0845
Epoch 113/128
768/768 [==============================] - 69s 90ms/sample - loss: 0.9624 - val_loss: 1.0073
Epoch 114/128
768/768 [==============================] - 70s 91ms/sample - loss: 0.9673 - val_loss: 1.0013
Epoch 115/128
768/768 [==============================] - 69s 90ms/sample - loss: 0.9802 - val_loss: 1.0403
Epoch 116/128
768/768 [==============================] - 69s 90ms/sample - loss: 0.9519 - val_loss: 1.0205
Epoch 117/128
768/768 [==============================] - 69s 90ms/sample - loss: 0.9659 - val_loss: 1.0810
Epoch 118/128
768/768 [==============================] - 69s 90ms/sample - loss: 0.9573 - val_loss: 1.1098
Epoch 119/128
768/768 [==============================] - 69s 90ms/sample - loss: 0.9586 - val_loss: 1.1032
Epoch 120/128
768/768 [==============================] - 71s 93ms/sample - loss: 0.9838 - val_loss: 1.0715
Epoch 121/128
768/768 [==============================] - 70s 92ms/sample - loss: 0.9708 - val_loss: 1.0302
Epoch 122/128
768/768 [==============================] - 70s 91ms/sample - loss: 0.9498 - val_loss: 1.0505
Epoch 123/128
768/768 [==============================] - 68s 89ms/sample - loss: 0.9578 - val_loss: 1.0461
Epoch 124/128
768/768 [==============================] - 69s 90ms/sample - loss: 0.9576 - val_loss: 1.0127
Epoch 125/128
768/768 [==============================] - 71s 93ms/sample - loss: 0.9646 - val_loss: 1.0600
Epoch 126/128
768/768 [==============================] - 70s 91ms/sample - loss: 0.9669 - val_loss: 1.0048
Epoch 127/128
768/768 [==============================] - 66s 87ms/sample - loss: 0.9607 - val_loss: 1.0247
Epoch 128/128
768/768 [==============================] - 70s 91ms/sample - loss: 0.9631 - val_loss: 1.0263
```

Out[16]: `<tensorflow.python.keras.callbacks.History at 0x7fde6e49dd10>`

Then we can make predictions using U-Net model

In [17]:
```
prediction_, *_ = unet.predict( gradient.reshape((1,)+gradient.shape+(1,)))
prediction_ = np.squeeze( prediction_ )
```

Now we can compare the ground truth and the prediction from unet

In [18]:
```
fig, axs = plt.subplots(1, 4)
axs[0].set_title('prediction')
axs[0].imshow(prediction_)
axs[1].set_title('ground truth')
axs[1].imshow(ground_truth)
axs[2].set_title('gradient')
axs[2].imshow(gradient)
ground_truth = (ground_truth-np.amin(ground_truth))/(np.amax(ground_truth)-np.amin(ground_truth)+1.0e-10)
prediction_ = (prediction_-np.amin(prediction_))/(np.amax(prediction_)-np.amin(prediction_)+1.0e-10)
diff = ground_truth - prediction_
axs[3].set_title('difference')
im = axs[3].imshow(diff)
divider = make_axes_locatable(axs[3])
cax = divider.append_axes('right', size='5%', pad=0.05)
fig.colorbar(im, cax=cax, orientation='vertical')
plt.rcParams["figure.figsize"] = (20,10)
plt.show()

print( f'MAE is {np.sum(np.abs(diff))/(512*512)}' )
```

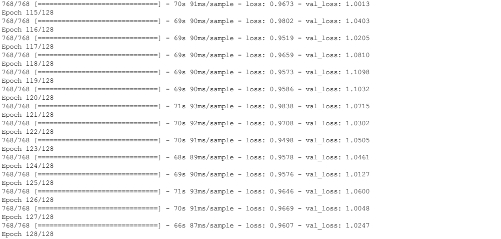


```
MAE is 0.08289105537011324
```

And the we can put the prediction results from MCNN and UNET together for a visual comparision

In [19]:
```
fig, axs = plt.subplots(1, 3)
axs[0].set_title('prediction from UNET')
axs[0].imshow(prediction_)
axs[1].set_title('prediction from MCNN')
axs[1].imshow(prediction)
axs[2].set_title('ground truth')
axs[2].imshow(ground_truth)
plt.rcParams["figure.figsize"] = (20,10)
plt.show()
```

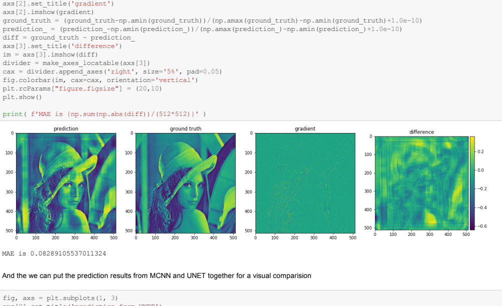

In [ ]:
